# Controlling Green‐Solvent Processability via the Dipole Moment of Nonfullerene Acceptor in Green‐Light Wavelength‐Selective Organic Solar Cells

**DOI:** 10.1002/cssc.202501902

**Published:** 2026-02-20

**Authors:** Shreyam Chatterjee, Naoya Tagashira, Naoto Shimohara, Yasuyuki Watanabe, Yutaka Ie

**Affiliations:** ^1^ The Institute of Scientific and Industrial Research (SANKEN) The University of Osaka Ibaraki Osaka Japan; ^2^ Department of Mechanical and Electrical Engineering Faculty of Engineering Suwa University of Science Chino Nagano Japan; ^3^ Innovative Catalysis Science Division Institute for Open and Transdisciplinary Research Initiatives (OTRI) The University of Osaka Suita Osaka Japan

**Keywords:** agrivoltaics, organic solar cells, semiconductors, sustainable chemistry, wavelength‐selectivity

## Abstract

In pursuit of agrivoltaics applications in greenhouses, green‐light wavelength‐selective organic solar cells (GLWS‐OSCs) have emerged as a promising technology that enables simultaneous energy harvesting and crop cultivation. For their practical large‐scale fabrication, the development of environmentally benign processing methods, along with achieving high photovoltaic performance, is essential. Herein, to investigate the impact of the dipole moment of nonfullerene acceptors on their suitability for green‐solvent processing, we developed a symmetric acceptor, BTz‐TT‐FA, possessing an intrinsically low dipole moment. BTz‐TT‐FA shows green‐light absorption with a maximum absorption wavelength at 531 nm. The ionization potential and electron affinity were determined to be 5.63 and 3.02 eV, respectively, indicating that BTz‐TT‐FA possesses appropriate energy levels as an acceptor for use with poly(3‐hexylthiophene) (P3HT) as the donor. The P3HT:BTz‐TT‐FA film shows a high green‐light wavelength‐selectivity factor of 0.77, thereby maintaining an adequate photosynthetic rate in strawberries. The P3HT:BTz‐TT‐FA‐based OSCs showed superior photovoltaic performance, achieving higher power conversion efficiency under *p*‐xylene‐processed conditions, compared to those processed with chlorobenzene. The dependence of the process solvent on film crystallinity, morphology, and miscibility was investigated. These findings highlight the critical role of dipole moment in facilitating green‐solvent processing and demonstrate a promising pathway toward the development of sustainable GLWS‐OSCs.

## Graphical abstract

For the environmentally friendly fabrication of green‐light wavelength‐selective organic solar cells (GLWS‐OSCs), a new nonfullerene acceptor, BTz‐TT‐FA, bearing a low dipole moment is designed and developed. GLWS‐OSCs based on poly(3‐hexylthiophene) (P3HT) and BTz‐TT‐FA showed enhanced photovoltaic performance under *p*‐xylene processing compared to chlorobenzene. The molecular properties, photosynthetic rate in strawberries, and blend film characteristics are systematically investigated.

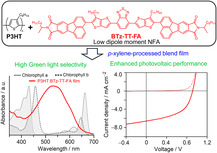

## 1 Introduction

In contrast to the conventional focus on maximizing power conversion efficiency (PCE), increasing attention has been directed toward the development of photovoltaic technologies with optical transparency, enabling the realization of semitransparent solar cells [1, 2, 3, 4]. Such transparency opens up unique applications, including integration into tandem architectures, building‐integrated windows, display technologies, and greenhouse roofs [5, 6, 7, 8, 9, 10, 11]. Organic solar cells (OSCs) are particularly promising in this regard, owing to their lightweight, flexibility, and most importantly, the tunability of their absorption and electronic properties through rational molecular design of organic semiconductor donors and acceptors [12, 13, 14, 15, 16, 17, 18, 19, 20, 21]. Among these, green‐light wavelength‐selective OSCs (GLWS‐OSCs) offer a compelling solution for agrivoltaics applications [22, 23]. Because photosynthetically active radiation essential for plant growth is predominantly absorbed in the blue (400–500 nm) and red (600–700 nm) regions of the solar spectrum, selective absorption of green light (500–600 nm) enables simultaneous energy harvesting and crop cultivation without significantly compromising photosynthetic efficiency. However, because GLWS‐OSCs absorb only ∼19% of the solar spectrum (500–600 nm), the available photocurrent is intrinsically limited compared with conventional solar cells that typically harvest photons across the 300–1400 nm range. Nevertheless, despite this limited power output, the generated electricity remains essential for environmental control in greenhouse agriculture. For practical implementation of GLWS‐OSCs in agrivoltaics, the integration of solar modules over hundreds of square meters of greenhouse roof is required. Therefore, for roll‐to‐roll manufacturing and large‐area fabrication, the use of nonhalogenated, environmentally benign solvents for the active layer becomes critical to meeting sustainability standards [24, 25, 26, 27, 28, 29].

**FIGURE 1:**
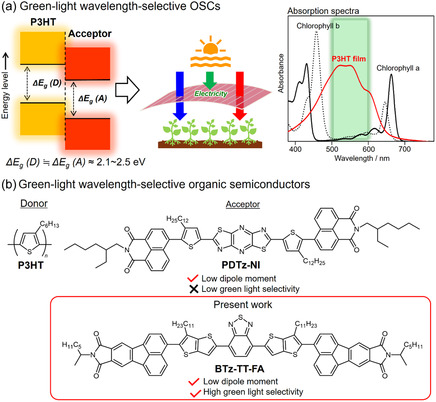
(a) Schematic diagram for GLWS‐OSCs and absorption spectra of chlorophylls a and b, and P3HT. (b) Chemical structures of green‐light wavelength‐selective NFAs.

Poly(3‐hexylthiophene) (P3HT) has been identified as a promising donor polymer for GLWS‐OSCs due to its low cost, large‐scale availability, and intrinsic green‐light‐selective absorption characteristics (Figure 1a) [30, 31, 32, 33, 34, 35, 36]. Recently, we have developed several nonfullerene acceptors (NFAs) compatible with P3HT [37, 38, 39, 40, 41, 42], demonstrating enhanced absorption selectivity in the green‐light region and offering dual functionality of power generation and photosynthesis. However, most of these systems have relied on halogenated solvents such as 1,2‐dichlorobenzene (*o*‐DCB), chlorobenzene (CB), and chloroform (CF) for device fabrication. To address this issue, we recently demonstrated that NFAs with low dipole moments are effective in controlling active‐layer morphology in P3HT‐based OSCs processed with nonhalogenated green solvents [43]. A highly symmetric pyradinodithiazole (PDTz)‐based acceptor (PDTz‐NI) showed a lower dipole moment than its asymmetric benzothiadiazole (BTz)‐based acceptor, resulting in improved miscibility with P3HT even in *o*‐xylene, a nonhalogenated solvent with lower polarity than CB. This improved compatibility enabled better morphological control in the active layer, leading to enhanced photovoltaic performance. These findings suggest that even when employing a BTz‐based core, the development of new green‐light‐selective NFAs is feasible through molecular design strategies that enhance molecular symmetry and reduce dipole moment. Based on this hypothesis, we designed a new acceptor molecule, BTz‐TT‐FA, which features a BTz core and fluoranthene imide terminal units [38, 39], bridged by a planar and symmetric thieno[3,2‐*b*]thiophene (TT) unit (Figure 1b). In this contribution, we report on the molecular design, synthesis, properties, and photovoltaic performance of BTz‐TT‐FA, along with its photosynthetic evaluation.

## 2 Results and Discussion

### 2.1 Theoretical Calculations

To investigate the electronic distribution of the molecular orbitals in the acceptors, theoretical calculations were performed using density functional theory (DFT) at the B3LYP/6‐31G(d, p) level. To reduce computational cost while retaining essential structural characteristics, the alkyl side chains were replaced with methyl groups. Theoretical calculations predict that PDTz‐NI and BTz‐TT‐FA possess nearly identical lowest unoccupied molecular orbital (LUMO) energy levels (−3.00 and −2.95 eV, respectively), whereas the highest occupied molecular orbital (HOMO) energy level is significantly upshifted from −5.88 eV for PDTz‐NI to −5.29 eV for BTz‐TT‐FA (Figure S1). Although both compounds showed spatial overlap between their HOMO and LUMO distributions, PDTz‐NI shows a more homogeneously delocalized HOMO, which contributes to its deeper HOMO energy level. In contrast, the HOMO of BTz‐TT‐FA is predominantly localized on the BTz‐TT unit. As a result, the higher‐lying HOMO level in BTz‐TT‐FA leads to an overall narrow band gap (*E*_g_) of 2.34 eV, compared to 2.88 eV for PDTz‐NI.

**FIGURE 2:**
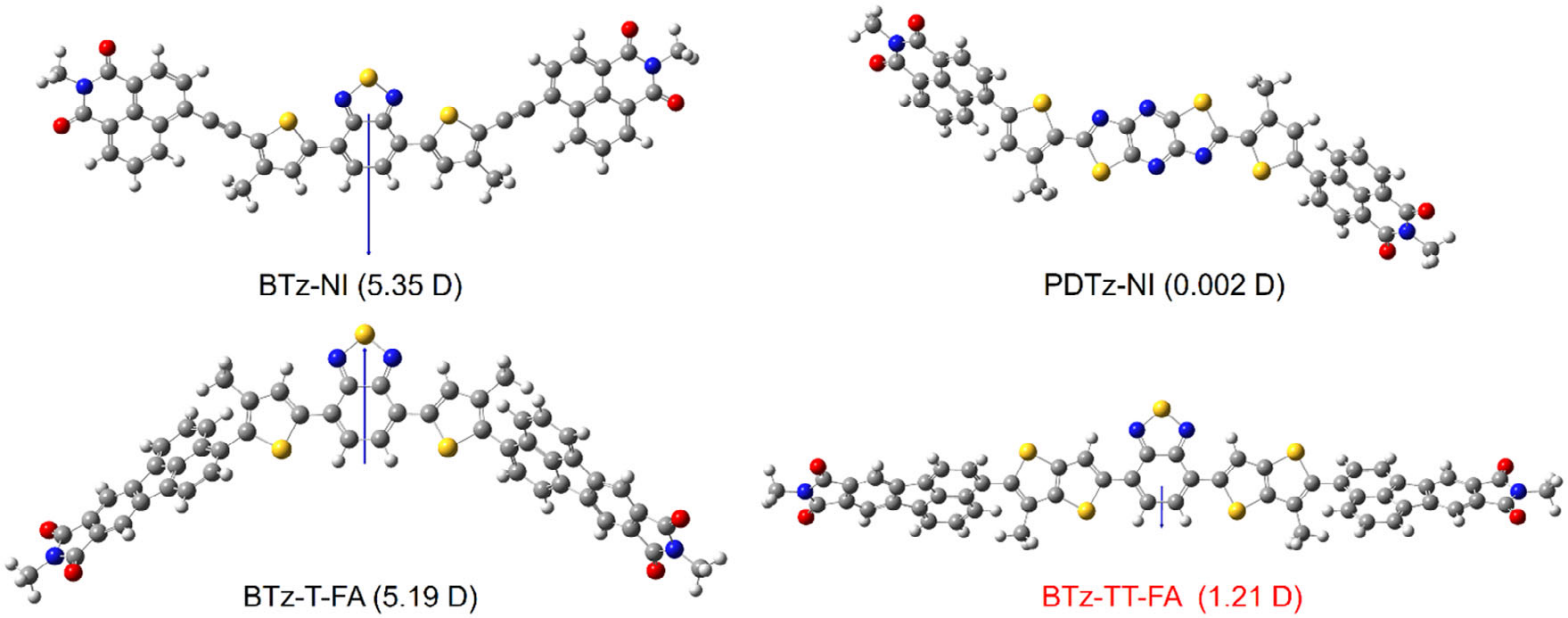
Optimized and energetically most stable structures of BTz‐NI, PDTz‐NI, BTz‐T‐FA, and BTz‐TT‐FA calculated at the B3LYP/6‐31 G(d,p) level. All alkyl groups were replaced with methyl groups to simplify the calculations. The length and orientation of the arrows represent the magnitude and direction of the overall dipole moment of the molecules.

We previously reported that the symmetric PDTz‐NI shows a lower dipole moment (*μ*) than BTz‐NI, which was attributed to the higher molecular symmetry of the PDTz unit [43]. Inspired by this, we designed a new molecule incorporating a symmetrical TT linker in the acceptor. Indeed, geometry optimization of the energetically most stable conformer of BTz‐TT‐FA showed a markedly reduced *μ* (1.21 D), in contrast to BTz‐T‐FA (5.35 D). (Figure 2). However, due to the presence of multiple conformers, thermal averaging was considered to reflect realistic *μ* behavior. Accordingly, ten possible conformers of BTz‐TT‐FA were calculated using DFT at the B3LYP/6‐31G (d, p) level [44]. The thermally averaged dipole moment (*μ*_ave_) was calculated using Equation (1), where *μ* *_i_*, *E* *_i_*, *k*_B_, and *T* are the dipole moment, energy of the *i*th metastable state, the Boltzmann constant, and temperature, respectively [45]. We set *T* to 300 K. The conformers and their associated energies and dipole moments are provided in Figures S2‐S3. Based on these calculations, the *μ*_ave_ values of BTz‐T‐FA and BTz‐TT‐FA were determined to be 4.31 D and 1.68 D, respectively. This result indicates that the newly designed BTz‐TT‐FA has a significantly lower *μ*_ave_.

(1) $${\left(\mu_{\mathrm{ave}}\right)}^{2}\;=\;[\Sigma_{i}\;{\left(\mu_{i}\right)}^{2}\;\exp\;(-E_{i}/k_{B}T)]/[\Sigma_{i}\;\exp\;(-E_{i}/k_{B}T)]$$

To estimate the absorption of BTz‐TT‐FA, time‐dependent (TD) DFT calculations were performed at the B3LYP/6‐31G(d, p) level. The results predicted that the transitions from the HOMO to LUMO occur at a wavelength of 531 nm with an oscillator strength (*f*) of 2.20 (Figure S4). These properties make BTz‐TT‐FA a promising NFA candidate for application in GLWS‐OSCs.

### 2.2 Photophysical and Electrochemical Properties

**FIGURE 3:**
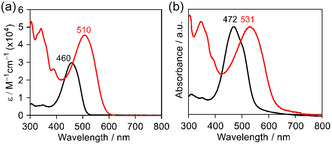
UV–vis absorption spectra of PDTz‐NI (black) and BTz‐TT‐FA (red) (a) in CF solutions and (b) in films.

The UV–vis absorption spectra of BTz‐TT‐FA along with PDTz‐NI in CF solution are shown in Figure 3a. BTz‐TT‐FA shows a strong absorption band with a maximum absorption wavelength at 510 nm, which is red‐shifted by 50 nm relative to PDTz‐NI. The molar extinction coefficient (*ε*) of BTz‐TT‐FA was determined to be 4.5 × 10^4^ M^–1^ cm^–1^, approximately 1.5‐times higher than that of PDTz‐NI (3.0 × 10^4^ M^–1^ cm^–1^) [43], indicating its enhanced photo absorption characteristics (Figure 3a). In thin films (Figure 3b), BTz‐TT‐FA shows a red‐shifted maximum absorption wavelength at 531 nm compared to 472 nm for PDTz‐NI. Furthermore, the extended *π*‐conjugation introduced by the TT unit in BTz‐TT‐FA enables broad and continuous absorption across the green region (500–600 nm) of the visible spectrum. Based on the absorption onset in film, the optical energy gaps (Δ*E*_opt_) of BTz‐TT‐FA were determined to be 2.01 eV, which is nearly identical to that of the P3HT film (1.99 eV) [33].

**FIGURE 4:**
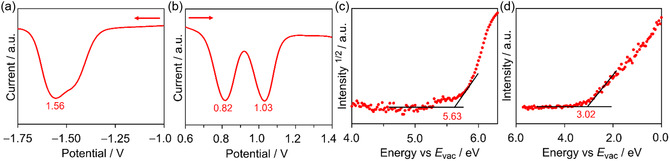
Differential pulse voltammograms of BTz‐TT‐FA in (a) negative and (b) positive sweeps. (c) PYS and (d) LEIPS spectra of the BTz‐TT‐FA films.

To investigate the electrochemical properties, differential pulse voltammetry measurements of BTz‐TT‐FA were performed in *o*‐DCB/acetonitrile (CH_3_CN) (10/1) solutions containing 0.1 M tetrabutylammonium hexafluorophosphate (TBAPF_6_) as the supporting electrolyte. The potentials were calibrated using the ferrocene/ferrocenium (Fc/Fc^+^) redox couple as an internal standard. The resulting voltammograms are shown in Figure 4. Based on the oxidation (*E*_ox_) and reduction (*E*_red_) potentials, and assuming that the energy level of Fc/Fc^+^ is 4.8 eV below the vacuum level, the HOMO/LUMO energy levels of BTz‐TT‐FA were estimated to be −5.62/−3.24 eV (Figure 4a, b).These experimentally determined values are in good qualitative agreement with those obtained from theoretical calculations (Figure S1). Moreover, the energy‐level alignment between BTz‐TT‐FA (LUMO: −3.02 eV) and P3HT (LUMO: −2.81 eV) provides a sufficient driving force for efficient charge separation. The HOMO energy level of BTz‐TT‐FA also shows a sufficiently large offset relative to that of P3HT (HOMO: −4.72 eV), providing an adequate driving force for exciton dissociation through hole transfer from BTz‐TT‐FA to P3HT. We also evaluated the ionization potential (IP) and electron affinity (EA) of BTz‐TT‐FA films using photoelectron yield spectroscopy (PYS) and low‐energy inverse photoemission spectroscopy (LEIPS) measurements. Based on the onset of the spectra, the IP and EA values were determined to be 5.63 and 3.02 eV, respectively (Figure 4c,d).

### 2.3 Green‐Light Utilization Efficiencies and Photosynthetic Evaluation

**FIGURE 5:**
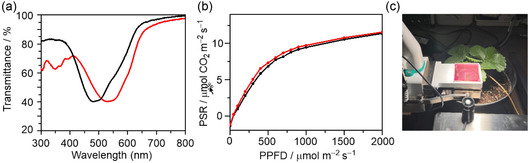
(a) Transmittance spectra of P3HT:PDTz‐NI (black) and P3HT:BTz‐TT‐FA (red) blend films. (b) PSR of strawberries under P3HT:PDTz‐NI (black) and P3HT:BTz‐TT‐FA (red) films against PPFD. These spectra are averages of three measurements. (c) Photo of photosynthetic experiments with the P3HT:BTz‐TT‐FA film.

The green light wavelength selectivity factor (*S*_G_) is a quantitative metric that evaluates how selectively a GLWS‐OSC blend film absorbs light in the 500–600 nm region relative to its absorption or transmission across the full visible spectrum (400–700 nm) [23, 40]. An *S*_G_ value of zero indicates no wavelength preference, i.e., uniform transmission across the visible range. From the transmittance spectra of the P3HT:BTz‐TT‐FA and P3HT:PDTz‐NI blend films (Figure 5a), the *S*_G_ values were calculated to be 0.77 and 0.51, respectively. The higher *S*_G_ value for the P3HT:BTz‐TT‐FA film indicates superior green‐light selectivity, primarily due to its increased transmittance in the blue region (Figure S5). Notably, the *S*_G_ value of the P3HT:BTz‐TT‐FA film exceeds those of representative benchmark systems based on P3HT, such as P3HT:*o*‐IDTBR (*S*_G_ = 0.26) and P3HT:ZY‐4CL (*S*_G_ = 0.21), highlighting the potential of BTz‐TT‐FA as a green‐light wavelength‐selective NFAs [40].

To evaluate the suitability of the P3HT:BTz‐TT‐FA blend film for GLWS‐OSCs, we conducted gas‐exchange measurements to assess the photosynthetic rate (PSR) of strawberry leaves under artificial sunlight [23, 40, 41]. During the experiment, the chamber temperature and humidity were maintained at 25°C and 50% relative humidity. The relationship between the PSR and the photosynthetic photon flux density (PPFD) is shown in Figure 5b, while the reproducibility data are provided in Tables S1 and S2. A corresponding image of the experimental setup is shown in Figure 5c. The photosynthetic activity observed under the P3HT:BTz‐TT‐FA film was slightly higher than that under the P3HT:PDTz‐NI film. This improved performance is attributed to the higher *S*_G_ value (0.77) of the P3HT:BTz‐TT‐FA film. These results highlight the importance of optimizing spectral transmittance to enhance photosynthetic efficiency and demonstrate that the P3HT:BTz‐TT‐FA film is a promising candidate for GLWS‐OSC applications in agrivoltaic systems.

### 2.4 OSC Characteristics

**FIGURE 6:**
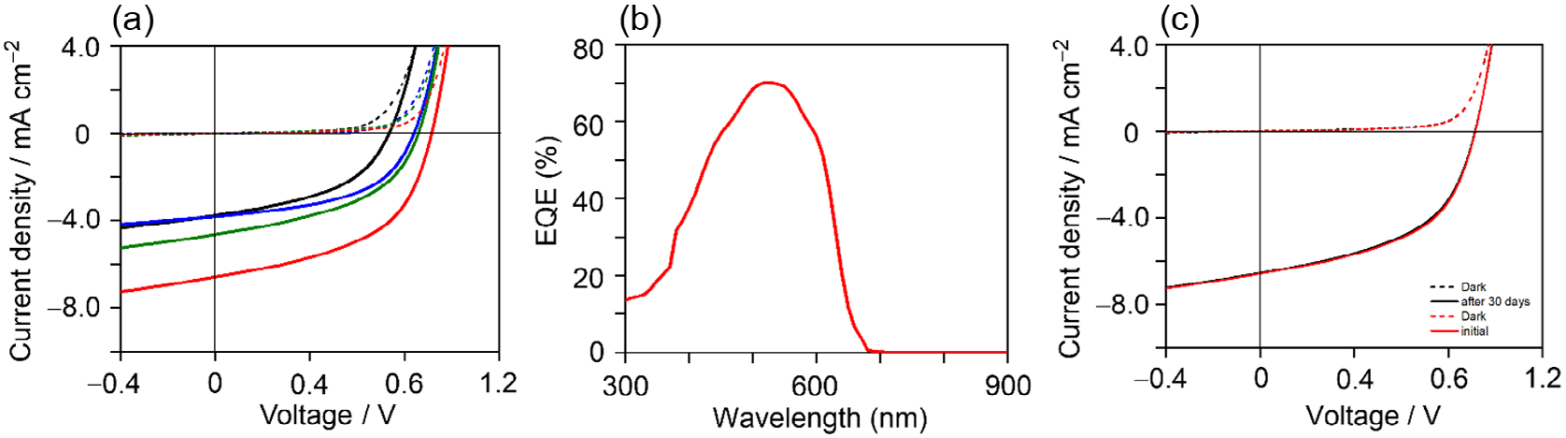
(a) *J*–*V* curves of the P3HT:BTz‐TT‐FA‐based OSCs films processed by CB (black), *o*‐xylene (blue), *p*‐xylene (green), and *p*‐xylene+BA (red). (b) EQE spectrum of P3HT:BTz‐TT‐FA‐based OSCs films processed by *p*‐xylene+BA. (c) Stability test of P3HT:BTz‐TT‐FA‐based OSCs films processed by *p*‐xylene+BA.

The bulk heterojunction OSCs were fabricated using an inverted device architecture, which offers enhanced environmental stability and simplified processing compared to conventional structures [31, 46]. The device configuration was glass/ITO/ZnO/P3HT:BTz‐TT‐FA (1:1 ratio)/poly(3,4‐ethylenedioxythiophene):poly(styrenesulfonate) (PEDOT:PSS)/Ag. Notably, with consideration for future large‐scale fabrication, solution‐processable PEDOT:PSS was employed as an alternative hole‐transporting layer instead of the commonly used MoO_3_. The current density (*J*)–voltage (*V*) curves of the best‐performing devices are shown in Figure 6a, and the corresponding photovoltaic parameters are summarized in Table 1. All *J*–*V* curves are summarized in Figure S6, and the statistical photovoltaic parameters are summarized in Tables S3‐S6. To evaluate the compatibility of different processing solvents for the P3HT:BTz‐TT‐FA‐based OSCs, CB, *o*‐xylene, and *p*‐xylene were selected as representative halogenated and nonhalogenated green solvents.

**TABLE 1:** Photovoltaic characteristics for P3HT:BTz‐TT‐FA‐based OSCs.

Solvent	*J*_sc_/mA cm^−2^	*V*_oc_/V	FF	PCE/%
CB	3.77 (± 0.03)	0.74 (± 0.01)	0.46 (± 0.04)	1.28 (± 0.02)
*o*‐xylene	3.82 (± 0.11)	0.84 (± 0.01)	0.51 (± 0.09)	1.63 (± 0.02)
*p*‐xylene	4.64 (± 0.05)	0.86 (± 0.01)	0.47 (± 0.05)	1.85 (± 0.02)
*p*‐xylene + BA	6.59 (± 0.03)	0.92 (± 0.01)	0.51 (± 0.06)	3.05 (± 0.02)

The P3HT:BTz‐TT‐FA film showed typical photovoltaic behavior, confirming that the newly developed BTz‐TT‐FA functions as an acceptor. The PCE of the *o*‐xylene‐processed OSCs (1.63%) was clearly higher than that of the CB‐processed OSCs (1.28%). A similar trend, in which *o*‐xylene outperforms CB as the process solvent, was also observed for PDTz‐NI [43]. To further evaluate the compatibility of the P3HT:BTz‐TT‐FA blend with the processing solvent of lower dipole moment, *p*‐xylene, which has a dipole moment of zero and a boiling point comparable to that of *o*‐xylene, was employed. Interestingly, the PCE further improved to 1.85%, mainly due to an increase in short‐circuit current density (*J*_sc_). To achieve additional performance enhancement, 5 vol% of 4‐bromoanisole (BrA) was introduced as an effective additive [47, 48] into the *p*‐xylene‐processed P3HT:BTz‐TT‐FA blend. As a result, the PCE was notably improved to 3.05% at a P3HT:BTz‐TT‐FA ratio of 1:1.5. External quantum efficiency (EQE) measurements showed that the P3HT:BTz‐TT‐FA OSCs showed photoresponses in the green‐light region between 400 and 650 nm, with a maximum EQE of approximately 70% at 530 nm (Figure 6b). The *J*_sc_ value estimated from the EQE spectrum was 6.87 mA cm^−2^, showing a deviation of 4.41% from that obtained from the *J*−*V* measurements, confirming the reliability of the photovoltaic measurements. The long‐term stability of the P3HT:BTz‐TT‐FA‐based OSCs was also evaluated by monitoring the *J*−*V* characteristics over 30 days under N_2_‐filled storage conditions. As shown in Figure 6c and Table S7, the devices exhibited excellent stability, with a PCE loss of less than ∼3.0%. This robustness highlights the long‐term stability of the P3HT:BTz‐TT‐FA films, which is essential for agrivoltaic applications. Additionally, preliminary operational stability measurements under continuous illumination for 8 h were conducted. The corresponding *J*–*V* curves are provided in Figure S7, and the statistical photovoltaic parameters are summarized in Table S8. Notably, the device exhibited no observable performance degradation over the test period, indicating adequate operational stability.

To further evaluate the photovoltaic performance of the devices for agrivoltaics applications, we calculated the power conversion efficiency in the green‐light region (PCE‐GR) as a benchmark for energy utilization specifically within the green spectral range [23, 40]. Based on the *J*−*V* characteristics and EQE spectrum (Figure 6b), the PCE‐GR of the P3HT:BTz‐TT‐FA‐based OSCs was determined to be 10.2%, which lies at the higher end of the reported range for OSC systems processed with nonhalogenated green solvent. In contrast, the PCE‐GR value of the P3HT:PDTz‐NI‐based OSCs was only 7.4% [43].

### 2.5 Film Evaluation

**FIGURE 7:**
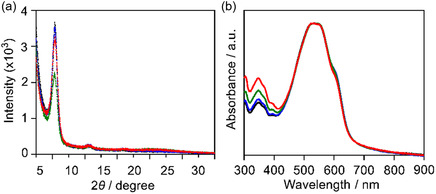
(a) Out‐of‐plane XRD and (b) UV–vis absorption spectra of P3HT:BTz‐TT‐FA films processed by CB (black), *o*‐xylene (blue), *p*‐xylene (green), and *p*‐xylene+BA (red).

To elucidate the correlation between solvent‐processing conditions and film crystallinity, X‐ray diffraction (XRD) measurements were conducted for P3HT:BTz‐TT‐FA films processed with CB, *o*‐xylene, *p*‐xylene, and *p*‐xylene with BA (abbreviated as *p*‐xylene+BA) (Figure 7a). All blend films exhibited a diffraction peak at around 2*θ* ≈ 5°, which is attributed to the crystalline structure of P3HT [49]. The intensity of this peak decreased in the order of CB‐, *o*‐xylene‐, *p*‐xylene‐, and *p*‐xylene+BA‐processed films, suggesting that the crystallinity of the blend films decreases in the same sequence. In the thin‐film absorption spectrum of P3HT, a shoulder peak around 650 nm is known to appear, which is associated with enhanced interchain ordering and stronger excitonic coupling [50]. Therefore, the shoulder peaks of these blend films were also compared. As shown in Figure 7b, the blend films processed from CB and *o*‐xylene exhibited a more pronounced vibronic shoulder compared to those processed from *p*‐xylene and *p*‐xylene+BA. This trend is consistent with the crystalline behavior observed in the XRD measurements.

To further investigate the effect of solvent polarity on dipole moment of the acceptors and the BA additive, we performed photoluminescence quenching (PLQ) measurements (Figure S8). Acceptors with lower dipole moments, when processed from low‐polarity solvents such as *o*‐xylene, *p*‐xylene, and *p*‐xylene+BA, clearly exhibited stronger PL quenching than those processed using the high‐polarity solvent CB, indicating more efficient exciton dissociation at the donor–acceptor interfaces. Furthermore, incorporation of BA into the *p*‐xylene processing solvent resulted in enhanced PL quenching in the P3HT:BTz‐TT‐FA blend. Combined with the atomic force microscopy(AFM) and XRD results, this suggests that BA promotes finer nanoscale mixing and increases interfacial area without significantly disrupting molecular ordering. Consequently, the optimized morphology facilitates both charge generation and transport, leading to simultaneous improvements in *J*_sc_ and *V*_oc_ in BA‐processed devices.

**FIGURE 8:**
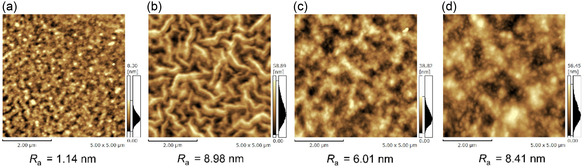
AFM height images of the P3HT:BTz‐TT‐FA film processed by (a) CB, (b) *o*‐xylene, (c) *p*‐xylene, and (d) *p*‐xylene+BA.

AFM measurements were performed on the P3HT:BTz‐TT‐FA films to investigate the influence of processing solvents on morphology. As shown in Figure 8, films processed from *o*‐xylene, *p*‐xylene, and *p*‐xylene+BA exhibited nanometer‐scale grain features with average roughness (*R*_a_) values of 8.98, and 6.01, nm, and 8.41nm respectively, whereas the CB‐processed film displayed a smooth surface with a *R*_a_ of 1.14 nm. AFM images of pristine P3HT processed from different solvents exhibit a similar trend to those of the P3HT:BTz‐TT‐FA blend films (Figure S9). Based on the XRD and AFM results, it is indicated that in CB, the high solubility of both P3HT (19 mg mL^−1^) and BTz‐TT‐FA (14 mg mL^−1^) leads to an overly homogeneous morphology, while still promoting P3HT crystallization. In contrast, the much lower solubility of P3HT (∼2 mg mL^−1^) and BTz‐TT‐FA (∼6 mg mL^−1^) in *o*‐xylene and *p*‐xylene promotes favorable aggregation, while simultaneously reducing P3HT crystallization.

Although such film properties would typically reduce the PCE in conventional P3HT‐based OSCs, an improvement in performance was observed in this study. To clarify the origin of this enhancement, the miscibility of P3HT and BTz‐TT‐FA was investigated. The Flory–Huggins interaction parameter (*χ*) was estimated from the surface free energies of the pristine films (Figure S10) [51]. A smaller *χ* value indicates stronger miscibility between P3HT and BTz‐TT‐FA. As summarized in Table S9, the P3HT:BTz‐TT‐FA films processed with *o*‐xylene (0.060) and *p*‐xylene (0.054) showed lower *χ* values than those processed with CB (0.063), indicating enhanced miscibility in the low‐polarity xylene solvents. Upon the addition of BrA, the *χ* value further decreased to 0.043, demonstrating its effectiveness in enhancing blend miscibility. Although the P3HT:BTz‐TT‐FA films fabricated from CB solutions exhibit a smooth morphology, the relatively low miscibility between P3HT and BTz‐TT‐FA implies the presence of aggregated P3HT domains, which is disadvantageous for efficient charge separation. In contrast, for the case of *o*‐xylene and *p*‐xylene‐processed blend films, the observed surface roughness reflects macroscopic phase separation, while good miscibility is still maintained at the nanoscale domain between P3HT and BTz‐TT‐FA, leading to the enhanced OSC performance. Thus, we conclude that the low dipole moment of BTz‐TT‐FA enhances its miscibility with P3HT and facilitates the formation of favorable phase‐separated nanostructures in less‐polar *p*‐xylene solvent. These factors are critical for efficient charge generation and transport in P3HT:BTz‐TT‐FA blends.

## 3 Conclusion

In summary, we successfully designed and synthesized a new NFA, BTz‐TT‐FA, with a low dipole moment, aimed at enabling the fabrication of GLWS‐OSCs using nonhalogenated green solvents. BTz‐TT‐FA exhibited strong absorption in the green‐light region (500–600 nm), and whereas PYS and LEIPS measurements confirmed that its energy levels are well aligned for efficient electron‐accepting behavior in OSCs. In addition, the film exhibited a high *S*_G_ value of 0.77. Notably, PSR measurements using strawberry leaves revealed enhanced performance under the P3HT:BTz‐TT‐FA film compared with the P3HT:PDTz‐NI film. When blended with P3HT, the resulting active layer showed superior device performance when processed with *o*‐xylene and *p*‐xylene compared with CB. Furthermore, optimization with BA as an additive led to promising improved GLWS‐OSC performance, achieving a PCE‐GR of 10.2%. Combined XRD, PLQ, AFM, and miscibility measurements revealed that although P3HT:BTz‐TT‐FA films showed relatively rough morphology under *o*‐xylene‐ and *p*‐xylene‐processed conditions, good miscibility was still attained at the nanoscale, which can be attributed to the intrinsically low dipole moment of BTz‐TT‐FA. These results reveal the decisive role of dipole moment in achieving green‐solvent processing for P3HT‐based OSCs and pave the way for the development of sustainable GLWS‐OSCs.

## Supporting Information

Additional supporting information can be found online in the Supporting Information section. **Supporting** **Fig.** **S1:** Energy levels and molecular orbitals of PDTz‐NI (left) and BTz‐TT‐FA (right) calculated at B3LYP/6‐31 G(d,p) level. All the alkyl groups were replaced with methyl groups to ease the calculation. **Supporting Fig. S2** **:** Optimized structures of the conformers for BTz‐T‐FA calculated at the B3LYP/6‐31 G(d,p) level. All alkyl groups were replaced with methyl groups to simplify the calculations. **Supporting Fig. S3** **:** Optimized structures of the conformers for BTz‐TT‐FA calculated at the B3LYP/6 31 G(d,p) level. All alkyl groups were replaced with methyl groups to simplify the calculations. **Supporting Fig. S4** **:** Simulated absorption spectrum (solid line) and oscillator strength (black circle with dropline) of BTz‐TT‐FA using TD‐DFT calculations at the B3LYP/6‐31 G(d,p) level. **Supporting Fig. S5** **:** Complementary absorption spectra of chlorophyll a and chlorophyll b along with the P3HT:BTz‐TT‐FA blend film. **Supporting** **Fig.** **S6:** Supplementary J–V curves of the (a) CB, (b) o‐xylene (c) *p*‐xylene and *p*‐xylene+ BA‐ processed P3HT:BTz‐TT‐FA devices. **Supporting Fig. S7** **:** J–V curves of the P3HT:BTz‐TT‐FA‐based OSCs films processed by *p*‐xylene+ BA (red) under continuous illumination. **Supporting** **Fig.** **S8:** PL spectra of P3HT:BTz‐TT‐FA‐based OSCs films processed by CB (black), o xylene (blue), *p*‐xylene (green), and *p*‐xylene+BA (red). For comparison, the PL spectrum of P3HT is shown as an orange line. **Supporting Fig. S9:** AFM height images of pristine BTz‐TT‐FA and P3HT films processed by (a) CB (b) o‐xylene, (c) *p*‐xylene, and (d) *p*‐xylene+BA. **Supporting** **Fig.** **S10:** Contact angle measurements of the pristine BTz‐TT‐FA and P3HT films processed from different solvents. **Supporting** **Table** **S1:** The photosynthetic data of the P3HT: BTz‐TT‐FA blend films. **Supporting Table S2:** The photosynthetic data of the P3HT:PDTz‐NI blend films. **Supporting Table S3** **:** OSCs based on CB‐processed P3HT:BTz‐TT‐FA blend films. **Supporting Table S4** **:** OSCs based on o‐xylene‐processed P3HT:BTz‐TT‐FA blend films. **Supporting Table S5:** OSCs based on *p*‐xylene‐processed P3HT:BTz‐TT‐FA blend films. **Supporting Table S6** **:** OSCs based on *p*‐xylene+BA‐processed P3HT:BTz‐TT‐FA blend films. **Supporting Table S7** **:** Stability of OSCs based on *p*‐xylene+BA‐processed P3HT:BTz‐TT‐FA blend films. **Supporting Table S8** **:** Photovoltaic data under continuous illumination of OSCs based on *p*‐xylene+BA processed P3HT:BTz‐TT‐FA blend films. **Supporting Table S9:** SFE characteristics of materials. The authors have cited additional references within the Supporting Information[52].

## Funding

Japan Society for the Promotion of Science (Grant 20KK0123, 23K04913, and 24H00482); Japan Science and Technology Corporation (Grant JPMJCR20R1, JPMJSP2138).

## Conflicts of Interest

The authors declare no conflicts of interest.

## Supporting information

Supplementary Material

## Data Availability

The data that support the findings of this study are available in the supplementary material of this article.
