# Supplementary material for: Controlling Green‐Solvent Processability via the Dipole Moment of Nonfullerene Acceptor in Green‐Light Wavelength‐Selective Organic Solar Cells
Source: ChemSusChem. 2026 Feb 20;19(4):e202501902. doi: 10.1002/cssc.202501902 (PMC12922472; doi:10.1002/cssc.202501902)
Supplement: Supplementary file 1 — Supplementary Material [file CSSC-19-e202501902-s001.pdf]

Supporting Information

Controlling Green-Solvent Processability via the Dipole Moment of Nonfullerene Acceptor in Green-Light Wavelength-Selective Organic Solar Cells

Shreyam Chatterjee, [1*] *Naoya Tagashira,* [1] *Naoto Shimohara,*[1] *Yasuyuki Watanabe,*[2] *Yutaka Ie*[1,3*]

[1] The Institute of Scientific and Industrial Research (SANKEN), The University of Osaka, 8-1 Mihogaoka, Ibaraki, Osaka 567-0041, Japan

[2] Department of Mechanical and Electrical Engineering, Faculty of Engineering, Suwa University of Science, 5000-1 Toyohira, Chino, Nagano 391-0292, Japan

[3]Innovative Catalysis Science Division, Institute for Open and Transdisciplinary Research Initiatives (OTRI), The University of Osaka, 2-1 Yamadaoka, Suita, Osaka 565-0871, Japan

Table of Contents

**Supplementary Figures and Tables**	**S2–S9**
**General Information**	**S10–S11**
**Synthesis**	**S11-S12**
**NMR Spectra**	**S13**
**Computational Details**	**S14–S31**

Supplementary Figures and Tables

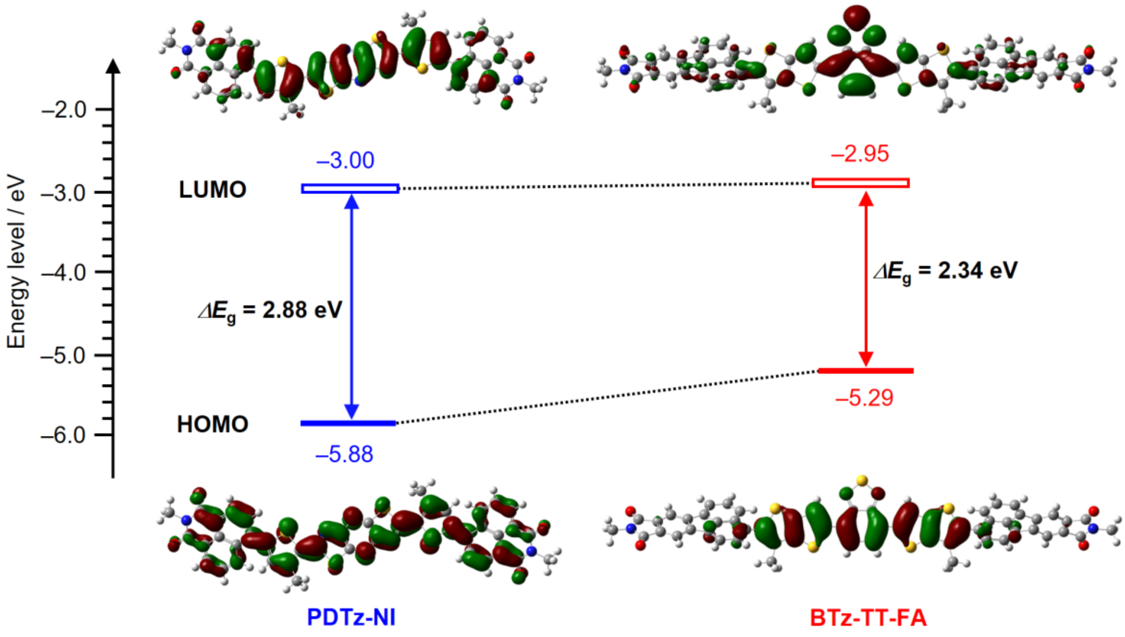

Figure S1. Energy levels and molecular orbitals of PDTz-NI (left) and BTz-TT-FA (right) calculated at B3LYP/6-31 G(d,p) level. All the alkyl groups were replaced with methyl groups to ease the calculation.

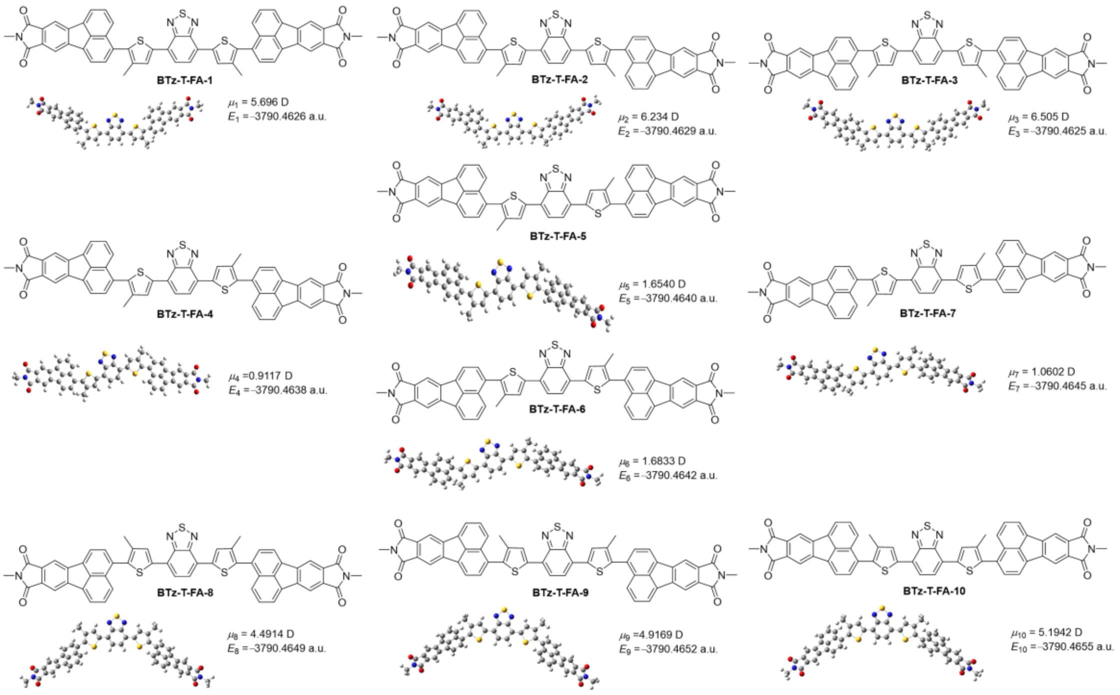

Figure S2. Optimized structures of the conformers for BTz-T-FA calculated at the B3LYP/6-31 G(d,p) level. All alkyl groups were replaced with methyl groups to simplify the calculations.

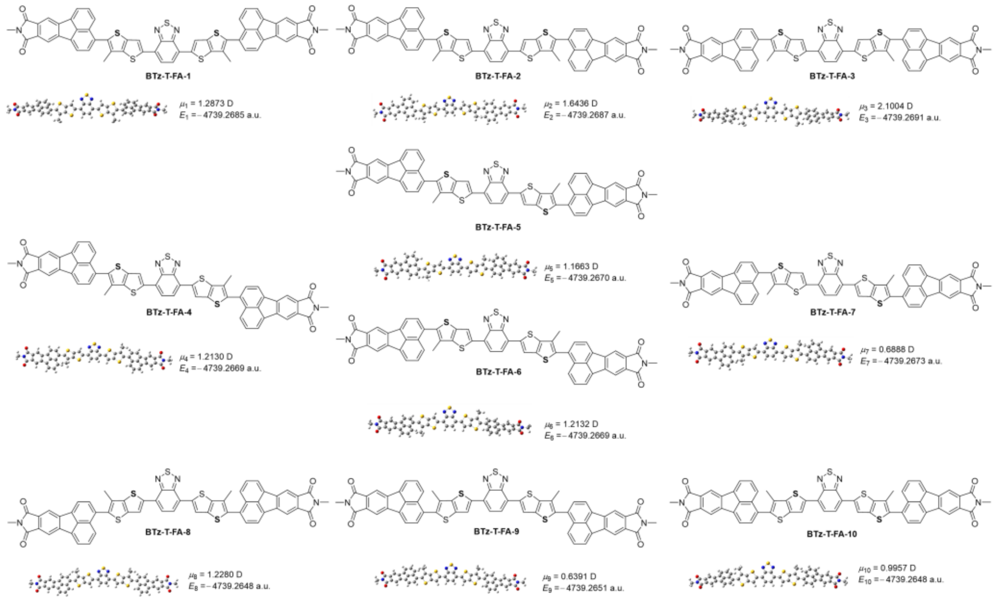

Figure S3. Optimized structures of the conformers for BTz-TT-FA calculated at the B3LYP/6-31 G(d,p) level. All alkyl groups were replaced with methyl groups to simplify the calculations.

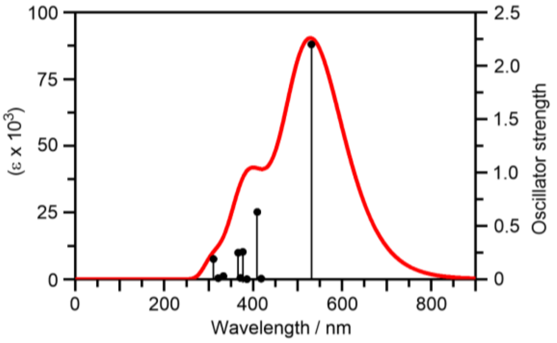

Figure S4. Simulated absorption spectrum (solid line) and oscillator strength (black circle with dropline) of BTz-TT-FA using TD-DFT calculations at the B3LYP/6-31 G(d,p) level.

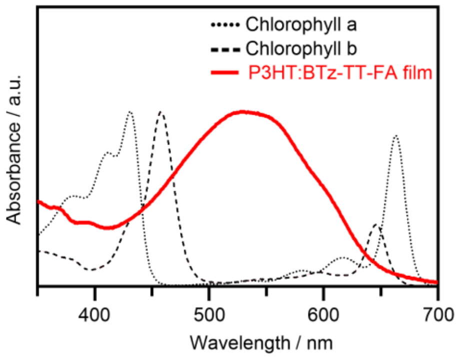

Figure S5. Complementary absorption spectra of chlorophyll *a* and chlorophyll *b* along with the P3HT:BTz-TT-FA blend film.

Table S1. The photosynthetic data of the P3HT: BTz-TT-FA blend films.

PPFD	Run 1	Run 2	Run 3
0	-1.20110472	-1.162125165	-1.261313494
50	0.264467625	0.295850255	0.208548189
100	1.342883947	1.368646483	1.432695558
200	3.601727271	2.924240491	3.562155235
300	5.403536244	4.511726935	5.230857673
400	6.755724532	6.260278006	6.628134812
500	7.367844307	7.368371482	7.270124694
600	8.229807237	8.26722767	7.622535415
700	8.817978417	8.99388103	8.300723618
800	9.282297849	9.445385964	8.620756555
900	9.727697135	10.00199319	8.848101071
1000	10.05470026	10.23983412	8.904175733
1500	10.94173627	11.25816225	10.16994743
2000	11.97764756	12.02075867	10.67077908

Table S2. The photosynthetic data of the P3HT:PDTz-NI blend films

PPFD	Run 1	Run 2	Run 3
0	-1.05937031	-1.508096803	-1.62408134
50	0.460536262	-0.059719963	0.169675809
100	1.234211093	0.689770879	0.947353688
200	3.395824483	2.405073521	2.613342971
300	5.363200737	4.151314494	3.80291471
400	6.799187854	5.333438357	5.392298898
500	7.70413938	6.164493726	6.593938523
600	8.97831544	6.979661637	7.332974476
700	9.27987366	7.486285636	7.75428467
800	10.16327728	7.934590694	8.275877321
900	10.41430284	8.25701861	8.865205672
1000	10.73759579	8.455916318	9.165460398
1500	12.05665242	9.218674486	10.39479682
2000	12.81271984	10.06428381	11.27950245

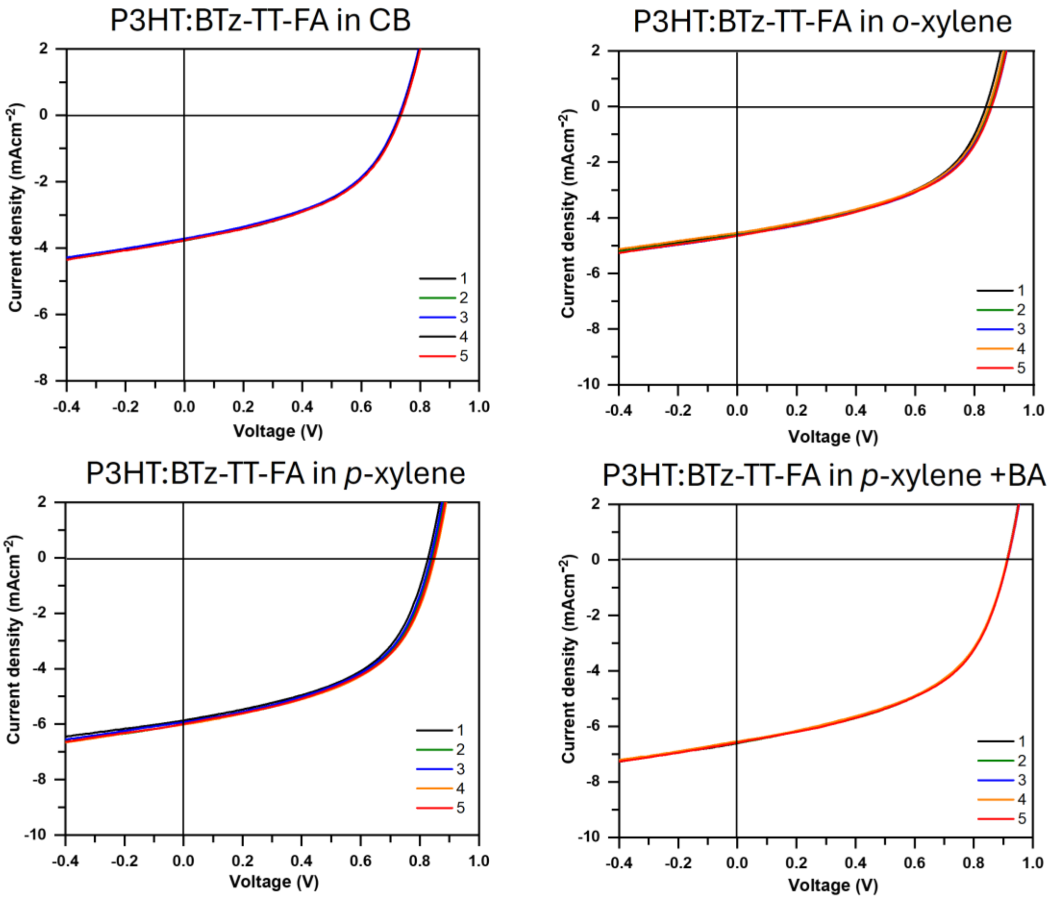

Figure S6. Supplementary *J–V* curves of the (a) CB, (b) *o*-xylene (c) *p*-xylene and *p*-xylene+ BA- processed P3HT:BTz-TT-FA devices.

Table S3. OSCs based on CB-processed P3HT:BTz-TT-FA blend films.

Run	J_{SC} / mA cm^{-2}	V_{OC} / V	FF	PCE / %
1	3.71	0.73	0.46	1.24
2	3.77	0.73	0.46	1.26
3	3.72	0.73	0.45	1.24
4	3.77	0.73	0.46	1.27
**5**	**3.77**	**0.74**	**0.46**	**1.28**
average	3.75 ± 0.03	0.73 ± 0.01	45 ± 0.04	1.26 ± 0.02

Table S4. OSCs based on *o*-xylene-processed P3HT:BTz-TT-FA blend films.

Run	J_{SC} / mA cm^{-2}	V_{OC} / V	FF	PCE / %
1	3.55	0.85	0.53	1.58
**2**	**3.82**	**0.84**	**0.51**	**1.63**
3	3.68	0.85	0.52	1.62
4	3.73	0.85	0.51	1.61
5	3.79	0.84	0.51	1.61
average	3.71 ± 0.11	0.85 ± 0.01	51 ± 0.09	1.61 ± 0.02

Table S5. OSCs based on *p*-xylene-processed P3HT:BTz-TT-FA blend films.

Run	J_{SC} / mA cm^{-2}	V_{OC} / V	FF	PCE / %
1	4.58	0.84	0.47	1.80
2	4.61	0.85	0.47	1.84
**3**	**4.64**	**0.86**	**0.47**	**1.85**
4	4.53	0.85	0.47	1.81
5	4.65	0.86	0.46	1.85
average	4.60 ± 0.05	0.85 ± 0.01	47 ± 0.05	1.83 ± 0.02

Table S6. OSCs based on *p*-xylene+BA-processed P3HT:BTz-TT-FA blend films.

Run	J_{SC} / mA cm^{-2}	V_{OC} / V	FF	PCE / %
1	6.61	0.91	0.50	3.02
2	6.60	0.91	0.51	3.05
3	6.56	0.92	0.51	3.05
4	6.54	0.91	0.50	3.01
**5**	**6.59**	**0.92**	**0.51**	**3.05**
average	6.58 ± 0.03	0.91 ± 0.01	51 ± 0.6	3.04 ± 0.02

Table S7. Stability of OSCs based on *p*-xylene+BA-processed P3HT:BTz-TT-FA blend films.

Run	J_{SC} / mA cm^{-2}	V_{OC} / V	FF	PCE / %
initial	**6.59**	**0.92**	**0.51**	**3.05**
5 days	6.56	0.91	0.50	3.00
10 days	6.49	0.92	0.50	2.99
15 days	6.56	0.91	0.51	3.02
20 days	6.54	0.91	0.50	3.00
30 days	6.55	0.91	0.50	3.01

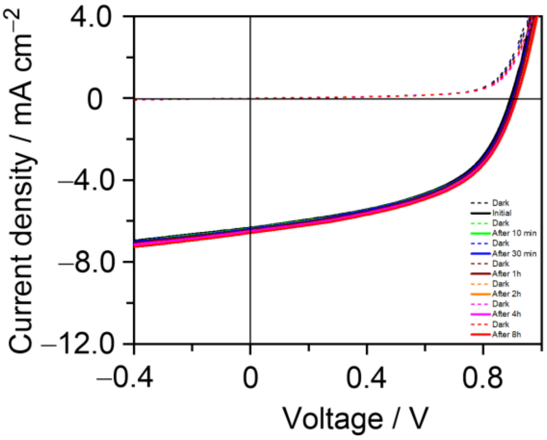

Figure S7. *J*–*V* curves of the P3HT:BTz-TT-FA-based OSCs films processed by *p*-xylene+ BA (red) under continuous illumination.

Table S8. Photovoltaic data under continuous illumination of OSCs based on *p*-xylene+BA-processed P3HT:BTz-TT-FA blend films.

Run	J_{SC} / mA cm^{-2}	V_{OC} / V	FF	PCE / %
1. Initial	6.326	0.893	0.503	2.841
2. After 10 min	6.363	0.902	0.503	2.888
3. After 30 min	6.388	0.901	0.504	2.902
4. After 1h	6.448	0.905	0.503	2.937
5. After 2h	6.557	0.914	0.499	2.991
6. After 4h	6.487	0.913	0.507	3.000
7. After 8h	6.585	0.914	0.507	3.053

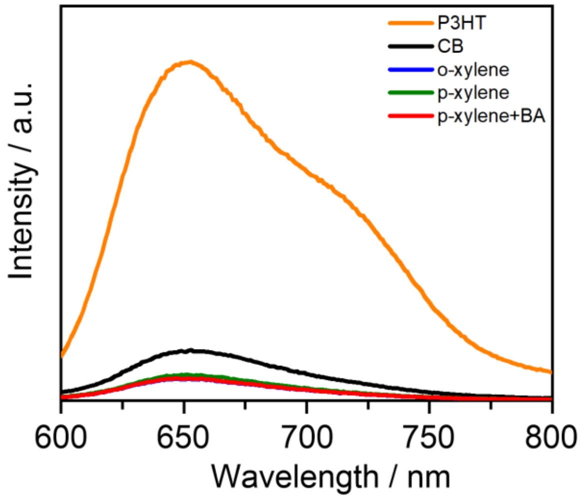

Figure S8. PL spectra of P3HT:BTz-TT-FA-based OSCs films processed by CB (black), *o*-xylene (blue), *p*-xylene (green), and *p*-xylene+BA (red). For comparison, the PL spectrum of P3HT is shown as an orange line.

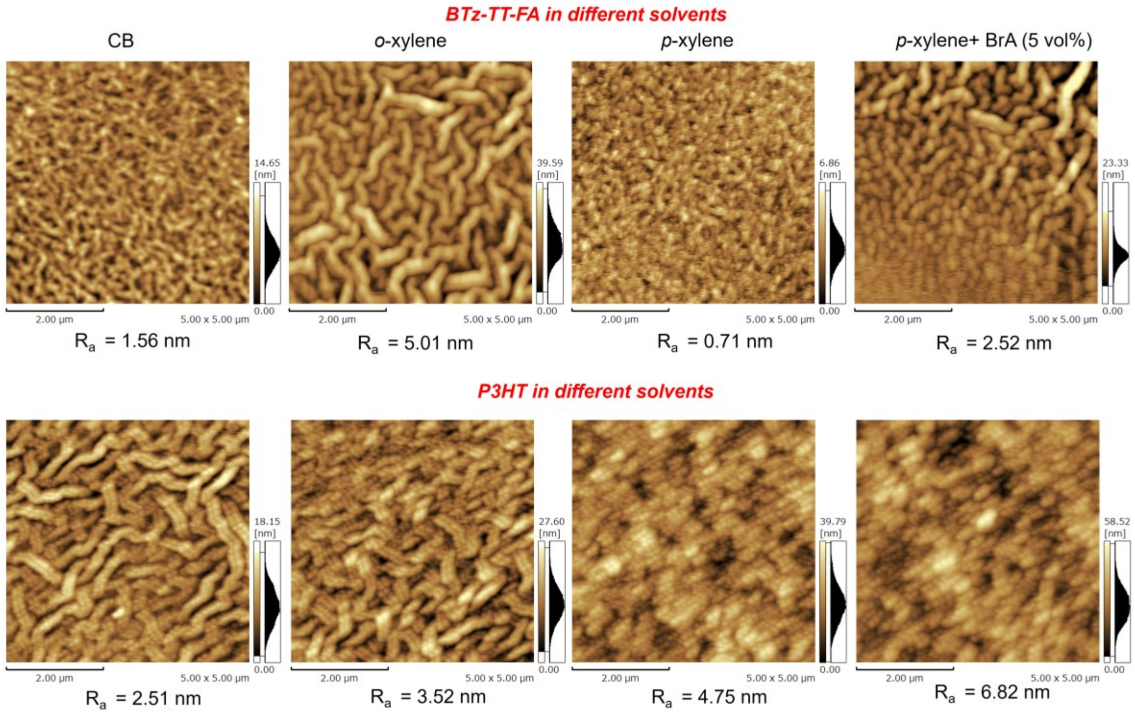

Figure S9. AFM height images of pristine BTz-TT-FA and P3HT films processed by (a) CB (b) *o*-xylene, (c) *p*-xylene, and (d) *p*-xylene+BA.

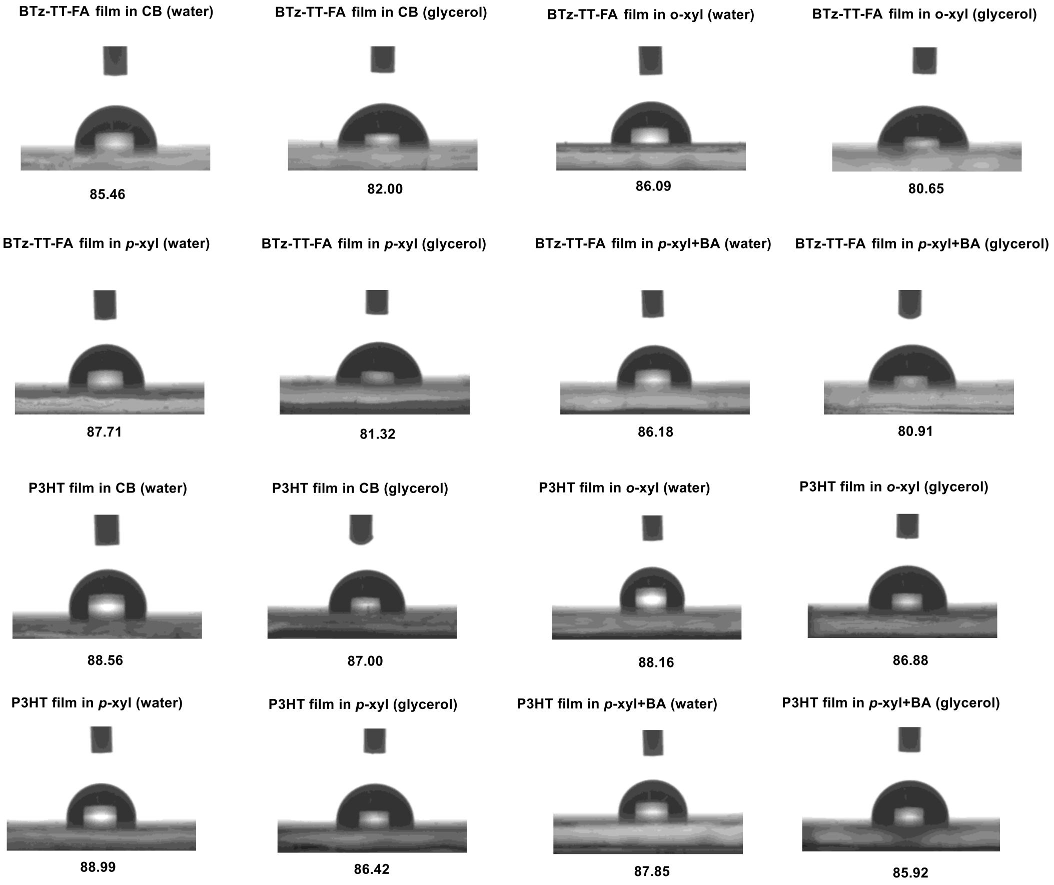

Figure S10. Contact angle measurements of the pristine BTz-TT-FA and P3HT films processed from different solvents.

Table S9. SFE characteristics of materials.

Materials	Process solvent	Contact angle / deg [a]	Contact angle / deg [b]	SFE / mJ cm^{-2}	γ_d / mJ cm^{-2}	γ_p / mJ cm^{-2}
**BTz-TT-FA**	CB	85.46	82.00	21.38	8.31	13.08
**BTz-TT-FA**	*o*-xyl	86.09	80.65	21.60	10.62	10.98
**BTz-TT-FA**	*p*-xyl	87.71	81.32	21.02	11.53	9.49
**BTz-TT-FA**	*p*-xyl+BA	86.18	80.91	21.47	10.38	11.09
**P3HT**	CB	88.56	87.00	19.13	5.72	13.4
**P3HT**	*o*-xyl	88.16	86.88	19.39	5.51	13.88
**P3HT**	*p*-xyl	88.99	86.42	18.91	6.66	12.25
**P3HT**	*p*-xyl+BA	87.85	85.92	19.60	6.21	13.39

[a] H_2O; [b] glycerol.

General Information

Column chromatography was performed on silica gel, KANTO Chemical silica gel 60N (40–50 μm). Thin-layer chromatography plates were visualized with UV light. Preparative GPC was performed on a Japan Analytical Industry LC-918 equipped with JAI-GEL 1H/2H. 1H and ^{13}C NMR spectra were recorded on a JEOL ECS-400 spectrometer in deuterated solvent with tetramethylsilane (TMS) as an internal standard. Data are reported as follows: chemical shift in ppm (δ), multiplicity (s = singlet, d = doublet, t = triplet, m = multiplet, br = broad), coupling constant (Hz). Mass spectra were obtained on a Shimadzu GCMS-QP-5050 or Shimadzu AXIMA-TOF. Elemental analysis was performed on a Perkin Elmer LS-50B instrument by the Elemental Analysis Section of Comprehensive Analysis Center (CAC), SANKEN, The University of Osaka.

UV-vis spectra were recorded on a Shimadzu UV-3600 spectrophotometer. Differential pulse voltammetry (DPV) were carried out on a BAS CV-620C voltammetric analyzer using a platinum disk as the working electrode, platinum wire as the counter electrode, and Ag/$AgNO_3$ as the reference electrode at a scan rate of 100 mV s^{-1}. Photoelectron yield spectroscopy (PYS) was performed by Bunkoukeiki BIP-KV202GD. Low-energy inverse photoemission spectroscopy (LEIPS) was performed by Ulvac-Phi, Inc. LEIPS system. The surface structures of the deposited organic films were observed by atomic force microscopy (AFM) (Shimadzu, SPM9600). X-ray diffraction (XRD) patterns were obtained using Bragg-Brentano geometry with CuKα radiation as an X-ray source with an acceleration voltage of 45 kV and a beam current of 200 mA. The scanning mode was set to 2θ–θ scans between 2°–30° with scanning steps of 0.01°.

Photovoltaic Device Fabrication: Organic photovoltaic devices were prepared with a structure of ITO/ZnO/P3HT:BTz-TT-FA/PEDOT:PSS/Ag. ITO-coated glass substrates were first cleaned by ultrasonication in toluene, acetone, water, and 2-propanol for 10 min each, respectively. ITO-coated glass substrates were then activated by ozone treatment for 1 h. ZnO was spin-coated on the ITO surface at 3500 rpm for 30 s and baked at 200 °C for 30 min. The active layers were then prepared by spin-coating on the ITO/ZnO electrode at 1000 rpm for 1 min in a glove box. For P3HT:BTz-TT-FA films, thermal annealing was performed at 140 °C for 10 min. The Ag electrode were evaporated on the top of active layer through a shadow mask to define the active area of the devices (0.06 cm^2) under a vacuum of 10^{-5} Pa to a thickness of 30, 100 nm determined by a quartz crystal monitor. After sealing the device from air, the photovoltaic characteristics were measured in air under simulated AM 1.5G solar irradiation (100 mW cm^{-2}) (SAN-EI ELECTRIC, XES-301S). The *J*–*V* characteristics of photovoltaic devices were

measured by using a KEITHLEY 2400 source meter. The EQE spectra were measured by using a Soma Optics Ltd. S-9240.

Photosynthetic rate evaluation: Photosynthetic rates were determined by gas exchange experiments using an Li-6800 photosynthesis system and a SOLAX series XC-100EF light source (SERIC Ltd., Japan). The light saturation point and photosynthetic rate were evaluated at a controlled temperature of 25 °C and a CO_2 concentration of 400 ppm.

Synthesis

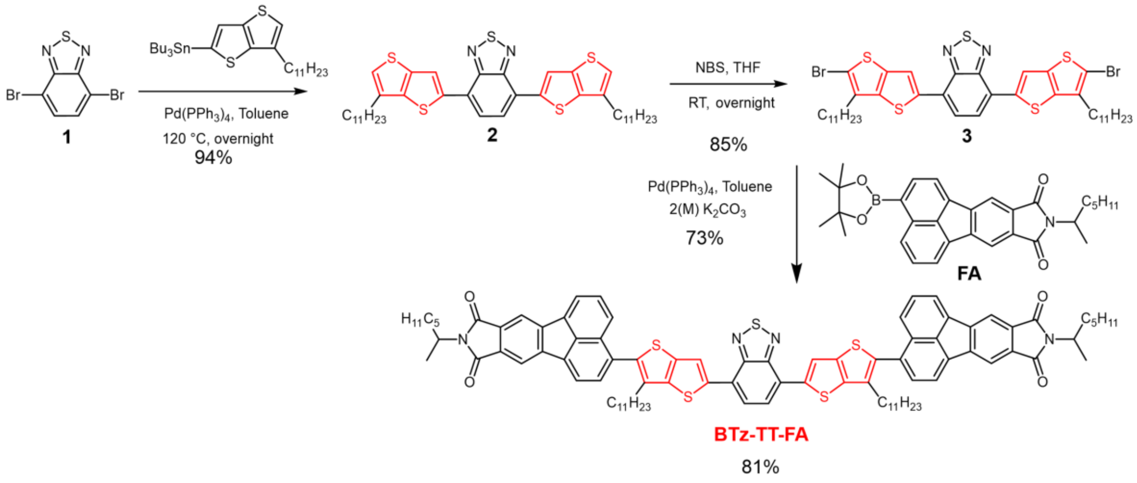

Synthetic scheme of **BTz-TT-FA**

Synthetic procedure:

Starting from 2,7-dibromobenzothiadiazole, the two-step route to compound **3** has been reported previously, and we followed a similar procedure.[52]

*Synthesis of **BTz-TT-FA***: $Pd(PPh_3)_4$ was added to a well-degassed glass vial equipped with a snap cap containing the solution of compound **3** (100 mg, 0.12 mmol) and **FA** (140 mg, 0.28 mmol) in toluene (10 mL). Aqueous solution of 2 mL of 2 M K_2CO_3 was added to the mixture, and the glass vial was purged with nitrogen, securely sealed, and heated at a temperature of 120 ºC for 12h. After cooling to room temperature, the reaction was quenched by the addition of H_2O and extracted with $CHCl_3$. After removal of the solvent under reduced pressure, the crude product was purified by column chromatography on silica gel (hexane/$CHCl_3$=2/1) followed by reprecipitation using $CHCl_3$ and methanol to give **BTz-TT-FA** as a red solid (136 mg, 81%). 1H-NMR (400 MHz, $CDCl_3$, δ) 8.55 (s, 2H), 8.35-8.34 (m, 4H), 8.16 (dd, J = 11.4, 6.9 Hz, 4H), 8.06 (d, J = 8.7 Hz, 2H), 7.97 (s, 2H), 7.82 (d, J = 7.3 Hz, 2H), 7.75 (dd, J = 8.2, 6.9 Hz, 2H),

4.40 (q, J = 7.5 Hz, 2H), 2.74 (t, J = 7.6 Hz, 4H), 2.15-2.09 (m, 2H), 1.79-1.69 (m, 6H), 1.52 (d, J = 6.9 Hz, 6H), 1.29-1.07 (m, 44H), 0.87-0.81 (m, 12H)

^{13}C NMR (100 MHz, $CDCl_3$, *δ*): 168.9, 152.7, 144.7, 144.1, 141.0, 140.5, 136.8, 135.2, 134.3, 133.7, 131.5, 129.8, 129.0, 126.5, 125.7, 122.7, 121.9, 120.9, 116.2, 77.4, 77.3, 77.1, 76.8, 47.7, 33.9, 32.0, 31.6, 29.8, 29.7, 29.5, 29.4, 29.2, 28.6, 26.6, 22.8, 22.6, 18.9, 14.2, 14.1; MS (MALDI) *m/z* 1455.6 (M^+, Calcd 1455.6); Anal. calcd. for $C_{92}H_{98}N_4O_4S_5$: C 74.46, H 6.66, N 3.68; found: C 74.25, H 6.64, N 3.68.

NMR Spectra

^{1}H NMR of **BTz-TT-FA** in $CDCl_3$

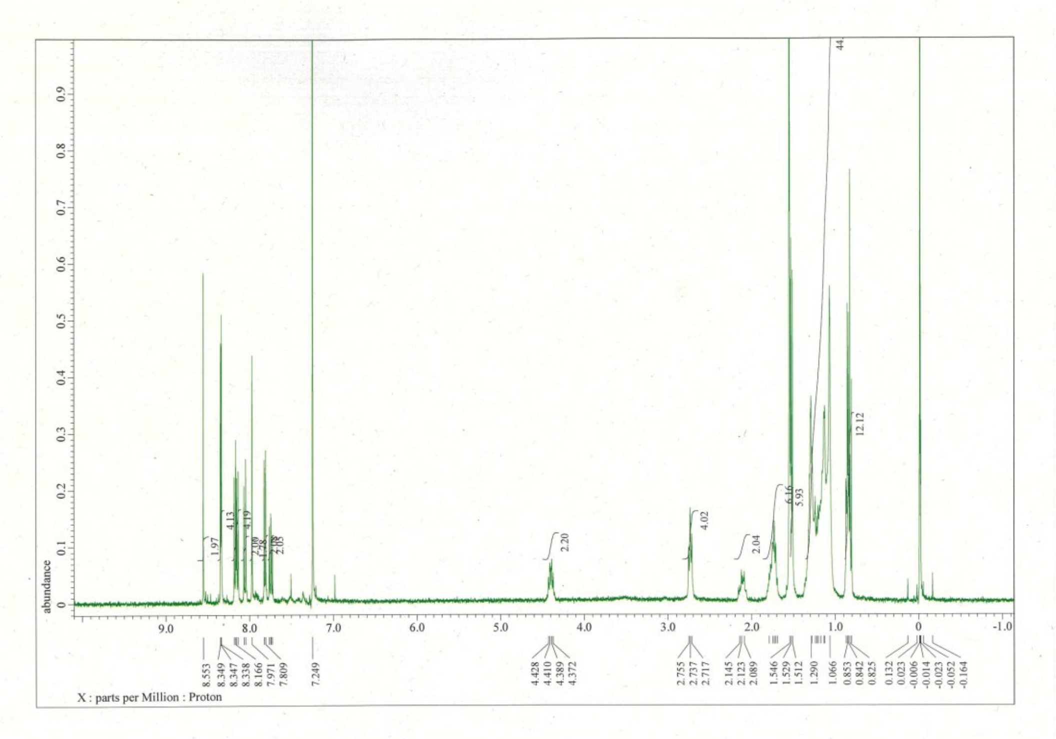

^{13}C NMR of **BTz-TT-FA** in $CDCl_3$

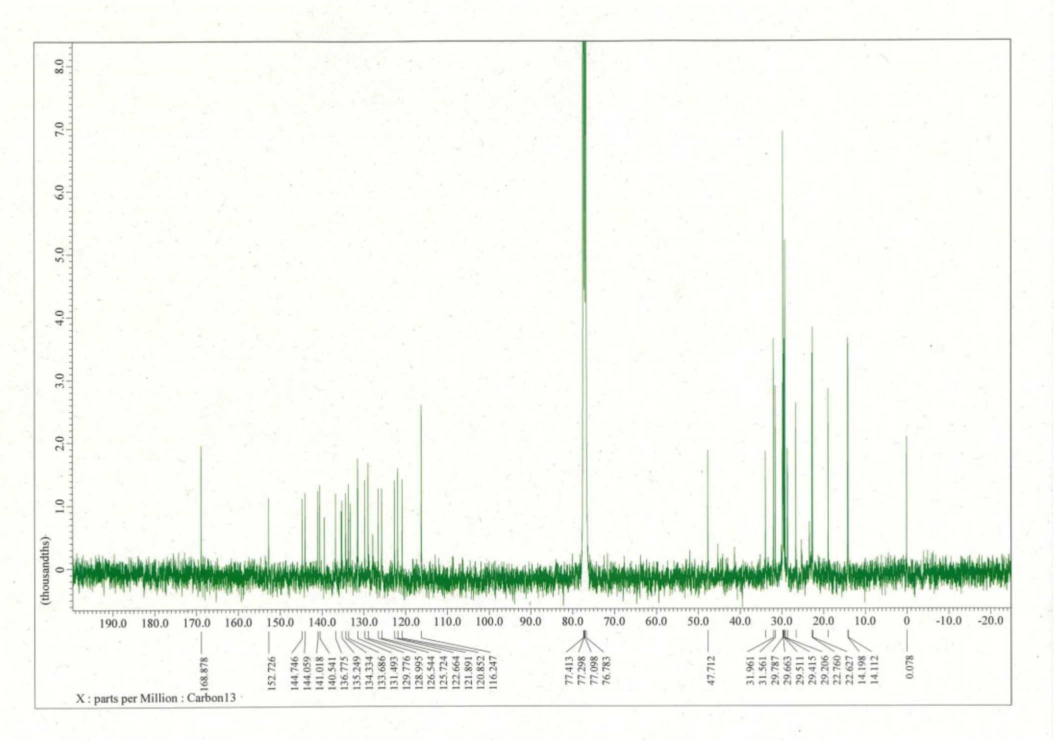

Computational Details

All calculations were conducted using Gaussian 09 program. The geometry was optimized with the restricted Becke Hybrid (B3LYP) at 6-31 G (d,p) level. The TD-DFT calculation was conducted at the CAM B3LYP/6-31G (d,p) level of theory. All the alkyl groups were replaced with methyl groups.

The calculated excited state of model compound for **BTz-TT-FA**

Excited State 1: Singlet-A 2.34 eV (531 nm) $f = 2.20$

HOMO > LUMO 0.64229

Excited State 3: Singlet-A 3.03 eV (409 nm) $f = 0.6290$

HOMO–3>LUMO+2 0.43848

Excited State 5: Singlet-A 3.29 eV (377 nm) $f = 0.2550$

HOMO-6 > LUMO+2 0.20992

HOMO-5 > LUMO+1 0.39092

HOMO-4 > LUMO 0.22665

HOMO > LUMO+2 0.13351

Excited State 7: Singlet-A 3.39 eV (366 nm) $f = 0.2469$

HOMO-5 > LUMO+1 0.19804

HOMO-4 > LUMO 0.16494

HOMO-2 > LUMO 0.54778

HOMO > LUMO+2 0.13119

Excited State 8: Singlet-A 3.72 eV (333 nm) $f = 0.0289$

HOMO-3 > LUMO 0.48557

HOMO-1 > LUMO 0.19162

HOMO-1 > LUMO+2 0.12159

Excited State 10: Singlet-A 3.99 eV (311 nm) $f = 0.1885$

HOMO-6 > LUMO+2 0.17400

HOMO-2 > LUMO+3 0.12131

HOMO-1 > LUMO+1 0.40959

HOMO > LUMO+2 0.20147

Optimized structure of **BTz-T-FA-1**

Center Number	Atomic Number	Atomic Type	Coordinates (Angstroms) X	Y	Z
1	6	0	-0.670151	3.729827	-0.022131
2	6	0	0.670165	3.729806	0.023702
3	6	0	1.413904	2.606611	0.049359
4	6	0	0.678957	1.469218	0.023639
5	6	0	-0.679060	1.469299	-0.022489
6	6	0	-1.414030	2.606714	-0.048067
7	7	0	1.172495	0.300147	0.040324
8	16	0	-0.000254	-0.927450	0.000541
9	7	0	-1.172733	0.300261	-0.039234
10	6	0	2.767322	2.694519	0.094974
11	6	0	-2.767345	2.694707	-0.093811
12	16	0	3.727814	1.593123	0.128943
13	6	0	4.834240	3.541773	0.168306
14	6	0	3.519043	3.801574	0.139728
15	6	0	5.061731	2.208423	0.123677
16	16	0	-3.727877	1.593495	-0.128016
17	6	0	-4.834279	3.542058	-0.167747
18	6	0	-3.519153	3.801858	-0.138845
19	6	0	-5.061764	2.208698	-0.123251
20	6	0	6.216270	1.479999	0.107208
21	6	0	-6.216322	1.480116	-0.107487
22	6	0	6.357762	0.125306	0.255731
23	6	0	7.598111	-0.392597	0.182265
24	6	0	8.717586	0.305766	-0.018850
25	6	0	8.612376	1.624549	-0.160836
26	6	0	7.378804	2.151658	-0.094683
27	6	0	5.449021	-0.848121	0.504246
28	6	0	5.752101	-2.155984	0.626464
29	6	0	7.011087	-2.605486	0.521003
30	6	0	7.948547	-1.681260	0.299445
31	6	0	-7.378982	2.151760	0.093158
32	6	0	-8.612780	1.624764	0.158181
33	6	0	-8.717850	0.306038	0.016225
34	6	0	-7.598229	-0.392402	-0.183374
35	6	0	-6.357612	0.125401	-0.255487
36	6	0	-7.948561	-1.681047	-0.300403
37	6	0	-7.010937	-2.605525	-0.520458
38	6	0	-5.751674	-2.156222	-0.624387
39	6	0	-5.448685	-0.848376	-0.502170
40	6	0	9.760876	-0.534813	-0.038443
41	6	0	9.279628	-1.777042	0.160905
42	6	0	10.084756	-2.849782	0.198879
43	6	0	11.392469	-2.608864	0.023507
44	6	0	11.869825	-1.370144	-0.175834
45	6	0	11.069191	-0.294990	-0.211913
46	6	0	-11.870442	-1.369570	0.169467
47	6	0	-11.392806	-2.608356	-0.028753
48	6	0	-10.084707	-2.849633	-0.200698
49	6	0	-9.279810	-1.776768	-0.163513
50	6	0	-9.761255	-0.534391	0.034903
51	6	0	-11.069650	-0.294613	0.207750
52	6	0	12.387640	-3.527626	0.023430
53	7	0	13.488272	-2.725545	-0.208715
54	6	0	13.216101	-1.376002	-0.322795
55	6	0	-13.216390	-1.375907	0.319444
56	7	0	-13.491598	-2.722116	0.177169
57	6	0	-12.387437	-3.527694	-0.024586
58	8	0	14.018063	-0.492529	-0.504664
59	8	0	12.389579	-4.724927	0.176186
60	8	0	-14.016120	-0.494941	0.522417
61	8	0	-12.386770	-4.727665	-0.154026

62	6	0	14.841355	-3.257792	-0.280193
63	6	0	-14.840965	-3.258524	0.279837
64	6	0	5.781242	4.712584	0.302124
65	6	0	-5.781255	4.712955	-0.301510
66	1	0	-1.126612	4.731784	-0.036209
67	1	0	1.126614	4.731656	0.037874
68	1	0	3.148121	4.834568	0.159587
69	1	0	-3.148083	4.834772	-0.158690
70	1	0	9.500921	2.248306	-0.350091
71	1	0	7.406830	3.226673	-0.287989
72	1	0	4.377945	-0.664949	0.645401
73	1	0	4.949903	-2.887730	0.829235
74	1	0	7.252824	-3.674008	0.626927
75	1	0	-7.407250	3.226850	0.285768
76	1	0	-9.501336	2.248709	0.346413
77	1	0	-7.252561	-3.674010	-0.626402
78	1	0	-4.949280	-2.888178	-0.825555
79	1	0	-4.377333	-0.665443	-0.641701
80	1	0	9.697590	-3.866344	0.363707
81	1	0	11.461461	0.720187	-0.373140
82	1	0	-9.697160	-3.866574	-0.362182
83	1	0	-11.461692	0.720320	0.370638
84	1	0	14.849976	-4.293805	-0.686749
85	1	0	15.483594	-2.646718	-0.953065
86	1	0	15.296261	-3.268446	0.736297
87	1	0	-15.598718	-2.524388	-0.074311
88	1	0	-14.964585	-4.170319	-0.346145
89	1	0	-15.063285	-3.519048	1.339537
90	1	0	6.526526	4.553917	1.113627
91	1	0	6.265414	4.961313	-0.669132
92	1	0	5.248037	5.640185	0.609465
93	1	0	-6.525027	4.555122	-1.114502
94	1	0	-6.267245	4.960322	0.669202
95	1	0	-5.247729	5.641163	-0.606463

Optimized structure of **BTz-T-FA-2**

Center Number	Atomic Number	Atomic Type	Coordinates (Angstroms) X	Y	Z
1	6	0	-0.696661	-3.347536	-0.277103
2	6	0	0.642251	-3.272805	-0.300729
3	6	0	1.325921	-2.114144	-0.223452
4	6	0	0.530059	-1.023480	-0.115384
5	6	0	-0.826578	-1.098867	-0.091209
6	6	0	-1.500078	-2.271206	-0.171127
7	7	0	0.959524	0.167393	-0.026581
8	16	0	-0.278111	1.323771	0.096139
9	7	0	-1.382980	0.036949	0.014666
10	6	0	2.683065	-2.129081	-0.251080
11	6	0	-2.847139	-2.433573	-0.152867
12	16	0	3.584180	-0.973688	-0.184752
13	6	0	4.782923	-2.881421	-0.355134
14	6	0	3.482020	-3.196191	-0.402069
15	6	0	4.931380	-1.564677	-0.106525
16	16	0	-3.865251	-1.390884	-0.041439
17	6	0	-4.867035	-3.390585	-0.175573
18	6	0	-3.539749	-3.577895	-0.211922
19	6	0	-5.164946	-2.072039	-0.106682
20	6	0	6.061291	-0.817473	-0.004268
21	6	0	-6.355576	-1.404565	-0.067468
22	6	0	7.259776	-1.292244	0.419883
23	6	0	8.337593	-0.505148	0.307252
24	6	0	8.319526	0.745459	-0.155140
25	6	0	7.141241	1.268787	-0.497698
26	6	0	6.050914	0.484980	-0.400317
27	6	0	7.521205	-2.435411	1.085575
28	6	0	8.765492	-2.798904	1.453922
29	6	0	9.829361	-2.009623	1.226031
30	6	0	9.587139	-0.819872	0.666304
31	6	0	-7.483312	-2.117098	-0.319809
32	6	0	-8.743059	-1.651519	-0.341991
33	6	0	-8.915482	-0.355405	-0.096384
34	6	0	-7.832270	0.382376	0.155804
35	6	0	-6.566121	-0.074404	0.185629
36	6	0	-8.248436	1.637940	0.373697
37	6	0	-7.359056	2.590448	0.663365
38	6	0	-6.077583	2.200919	0.728899
39	6	0	-5.707910	0.924085	0.504764
40	6	0	9.578314	1.206591	-0.132635
41	6	0	10.364745	0.233591	0.373380
42	6	0	11.685882	0.402701	0.534301
43	6	0	12.175495	1.592279	0.155653
44	6	0	11.392697	2.559774	-0.347846
45	6	0	10.070648	2.397893	-0.504190
46	6	0	-12.151738	1.160179	-0.109721
47	6	0	-11.737682	2.402877	0.184024
48	6	0	-10.442779	2.697869	0.370748
49	6	0	-9.583674	1.674626	0.247494
50	6	0	-10.001261	0.428197	-0.046705
51	6	0	-11.296562	0.134898	-0.234732
52	6	0	13.468853	1.988139	0.224276
53	7	0	13.382733	3.264622	-0.296772
54	6	0	12.109734	3.668116	-0.650734
55	6	0	-13.497155	1.107797	-0.254816
56	7	0	-13.840979	2.422516	-0.006182
57	6	0	-12.778688	3.266412	0.255240
58	8	0	11.795513	4.741678	-1.104007
59	8	0	14.468015	1.436767	0.617314
60	8	0	-14.251459	0.204932	-0.523969
61	8	0	-12.839345	4.450884	0.479170
62	6	0	14.541091	4.137722	-0.419409
63	6	0	-15.217197	2.893658	-0.062051
64	6	0	5.805379	-3.930762	-0.721758
65	6	0	-5.750534	-4.617310	-0.155640
66	1	0	-1.098689	-4.369925	-0.350524
67	1	0	1.150832	-4.245683	-0.384616
68	1	0	3.170222	-4.235504	-0.569491
69	1	0	-3.114175	-4.587752	-0.275330
70	1	0	7.072051	2.303126	-0.871349
71	1	0	5.133991	0.983977	-0.758428
72	1	0	6.702031	-3.095897	1.403259
73	1	0	8.914899	-3.751036	1.992022
74	1	0	10.839741	-2.302894	1.549881
75	1	0	-7.458127	-3.174362	-0.593699
76	1	0	-9.599347	-2.304307	-0.576565
77	1	0	-7.655233	3.633467	0.852412
78	1	0	-5.312989	2.956144	0.983982
79	1	0	-4.627449	0.788695	0.625975
80	1	0	12.328716	-0.385852	0.953389
81	1	0	9.428652	3.193174	-0.911668
82	1	0	-10.107299	3.717873	0.610826
83	1	0	-11.636876	-0.883258	-0.475693
84	1	0	15.471763	3.555346	-0.601842
85	1	0	14.432066	4.839857	-1.276040
86	1	0	14.667163	4.729959	0.515169
87	1	0	-15.932673	2.095714	0.237871
88	1	0	-15.383203	3.747244	0.632579

89	1	0	-15.461214	3.222024	-1.097810
90	1	0	6.764321	-3.500317	-1.082877
91	1	0	5.974803	-4.662198	0.099510
92	1	0	5.431887	-4.525084	-1.586973
93	1	0	-6.497169	-4.577752	0.669047
94	1	0	-6.227617	-4.794973	-1.145924
95	1	0	-5.168438	-5.541876	0.057354

Optimized structure of **BTz-T-FA-3**

Center Number	Atomic Number	Atomic Type	Coordinates (Angstroms) X	Y	Z
1	6	0	-0.706420	-2.999548	0.055767
2	6	0	0.701176	-2.998478	-0.067956
3	6	0	1.470676	-1.844185	-0.134179
4	6	0	0.723552	-0.619536	-0.033918
5	6	0	-0.723982	-0.621057	0.117296
6	6	0	-1.473687	-1.847183	0.165588
7	7	0	1.247964	0.608528	-0.060746
8	16	0	0.002570	1.667931	0.092767
9	7	0	-1.245390	0.605863	0.199274
10	6	0	2.918169	-1.888167	-0.301656
11	6	0	-2.921836	-1.894920	0.325746
12	16	0	3.960664	-0.492799	-0.101656
13	6	0	5.084049	-2.764476	-0.675604
14	6	0	3.684081	-2.997327	-0.608819
15	6	0	5.397612	-1.441673	-0.409746
16	16	0	-3.963914	-0.497075	0.143429
17	6	0	-5.090058	-2.781140	0.659368
18	6	0	-3.689057	-3.011000	0.602590
19	6	0	-5.402793	-1.453734	0.416692
20	6	0	6.707599	-0.766795	-0.432727
21	6	0	-6.713932	-0.780789	0.438374
22	6	0	7.798300	-1.206600	0.397510
23	6	0	9.005964	-0.501252	0.271323
24	6	0	9.201241	0.613100	-0.581187
25	6	0	8.133930	1.044110	-1.344603
26	6	0	6.905635	0.344822	-1.256083
27	6	0	7.789556	-2.252085	1.364357
28	6	0	8.931539	-2.542999	2.092965
29	6	0	10.146147	-1.825994	1.926489
30	6	0	10.180437	-0.793323	1.010709
31	6	0	-6.923439	0.311294	1.284688
32	6	0	-8.152960	1.008497	1.372847
33	6	0	-9.210362	0.594093	0.586812
34	6	0	-9.003745	-0.501132	-0.287561
35	6	0	-7.794198	-1.203243	-0.414162
36	6	0	-10.168410	-0.776552	-1.048535
37	6	0	-10.122867	-1.790209	-1.984833
38	6	0	-8.906691	-2.504802	-2.150070
39	6	0	-7.773804	-2.229166	-1.401594
40	6	0	10.592645	1.048788	-0.391244
41	6	0	11.194910	0.184997	0.585045
42	6	0	12.524905	0.352219	0.974973
43	6	0	13.224286	1.392119	0.373348
44	6	0	12.637245	2.234182	-0.577559
45	6	0	11.316288	2.086139	-0.983403
46	6	0	-12.645057	2.217943	0.571050
47	6	0	-13.218620	1.398713	-0.407544
48	6	0	-12.511157	0.372665	-1.023219
49	6	0	-11.187876	0.193502	-0.616090
50	6	0	-10.598678	1.035294	0.387016
51	6	0	-11.329563	2.060660	0.991053
52	6	0	14.637270	1.824370	0.567994
53	7	0	14.820911	2.920772	-0.287464
54	6	0	13.659852	3.227682	-1.013389
55	6	0	-13.671176	3.206099	1.009650
56	7	0	-14.820967	2.918732	0.259425
57	6	0	-14.625644	1.842254	-0.618811
58	8	0	13.560036	4.120969	-1.822276
59	8	0	15.482862	1.358849	1.291441
60	8	0	-13.578130	4.087385	1.827043
61	8	0	-15.456676	1.403985	-1.376198
62	6	0	16.066828	3.654591	-0.405939
63	6	0	-16.068471	3.649921	0.377654
64	6	0	6.067679	-3.839306	-1.061407
65	6	0	-6.076736	-3.866107	1.006911
66	1	0	-1.202470	-3.963917	0.057265
67	1	0	1.194926	-3.963142	-0.108554
68	1	0	3.252736	-3.968486	-0.825422
69	1	0	-3.258048	-3.985346	0.805136
70	1	0	8.211761	1.897394	-2.012240
71	1	0	6.080161	0.671250	-1.880793
72	1	0	6.878690	-2.813423	1.540879
73	1	0	8.896755	-3.341455	2.828145
74	1	0	11.013320	-2.092072	2.524103
75	1	0	-6.106101	0.623822	1.926946
76	1	0	-8.239840	1.846310	2.058704
77	1	0	-10.982519	-2.043506	-2.598642
78	1	0	-8.863107	-3.288741	-2.900251
79	1	0	-6.861308	-2.787996	-1.577565
80	1	0	13.002566	-0.288544	1.709476
81	1	0	10.883768	2.748994	-1.726189
82	1	0	-12.977779	-0.248926	-1.780926
83	1	0	-10.906664	2.707422	1.753292
84	1	0	16.893109	2.972949	-0.199383
85	1	0	16.141548	4.052626	-1.418875
86	1	0	16.107407	4.486183	0.305331
87	1	0	-15.843661	4.676519	0.670211
88	1	0	-16.577137	3.628392	-0.587136
89	1	0	-16.722169	3.201171	1.132877
90	1	0	7.023765	-3.417086	-1.376627
91	1	0	6.268558	-4.525932	-0.230550
92	1	0	5.673412	-4.439941	-1.887526
93	1	0	-7.037149	-3.452968	1.321033
94	1	0	-6.266407	-4.532000	0.156800
95	1	0	-5.691739	-4.486889	1.822471

Optimized structure of **BTz-T-FA-4**

Center Number	Atomic Number	Atomic Type	Coordinates (Angstroms) X	Y	Z
1	6	0	0.044546	-1.011336	-0.032527
2	6	0	-1.174387	-0.453586	0.019748
3	6	0	-1.386666	0.878369	0.081968
4	6	0	-0.243018	1.599922	0.083343
5	6	0	0.992854	1.038460	0.030996
6	6	0	1.188586	-0.300050	-0.029407
7	7	0	-0.209184	2.865505	0.131832
8	16	0	1.365702	3.500347	0.120399
9	7	0	1.925643	1.898943	0.043510
10	6	0	-2.645795	1.384774	0.135139

11	6	0	2.383788	-0.939735	-0.084141
12	16	0	-3.869057	0.578319	0.132478
13	6	0	-4.413095	2.748598	0.244190
14	6	0	-3.075855	2.648814	0.221198
15	6	0	-4.989162	1.527642	0.154912
16	16	0	3.711944	-0.331561	-0.089037
17	6	0	3.920675	-2.558313	-0.197846
18	6	0	2.614341	-2.256012	-0.162417
19	6	0	4.677212	-1.437584	-0.127898
20	6	0	-6.296909	1.136550	0.121172
21	6	0	6.028491	-1.238376	-0.113687
22	6	0	-6.797321	-0.134446	0.228369
23	6	0	-8.131202	-0.297934	0.148414
24	6	0	-9.022068	0.681113	-0.022717
25	6	0	-8.566175	1.926761	-0.126236
26	6	0	-7.236341	2.101119	-0.053132
27	6	0	-6.183840	-1.323424	0.440318
28	6	0	-6.826896	-2.505160	0.524921
29	6	0	-8.160296	-2.596833	0.415652
30	6	0	-8.814630	-1.448192	0.229450
31	6	0	6.824777	-2.332576	-0.003221
32	6	0	8.167159	-2.353224	0.038945
33	6	0	8.794981	-1.182316	-0.029522
34	6	0	8.050100	-0.080118	-0.137585
35	6	0	6.704819	-0.048095	-0.185903
36	6	0	8.890821	0.962734	-0.191337
37	6	0	8.406380	2.200797	-0.310606
38	6	0	7.071684	2.306577	-0.381439
39	6	0	6.266922	1.226608	-0.325999
40	6	0	-10.252778	0.152363	-0.058699
41	6	0	-10.122512	-1.178996	0.099321
42	6	0	-11.185637	-1.997584	0.112530
43	6	0	-12.380875	-1.409603	-0.043212
44	6	0	-12.508714	-0.082576	-0.201211
45	6	0	-11.449128	0.739143	-0.213036
46	6	0	12.358605	-0.933456	0.077680
47	6	0	12.419642	0.403764	-0.022726
48	6	0	11.318122	1.162789	-0.118848
49	6	0	10.149035	0.504276	-0.109816
50	6	0	10.089700	-0.837510	-0.008889
51	6	0	11.192239	-1.595214	0.088627
52	6	0	-13.585901	-2.027695	-0.060684
53	7	0	-14.431515	-0.953085	-0.258001
54	6	0	-13.807464	0.277052	-0.335029
55	6	0	13.594743	-1.479985	0.164669
56	7	0	14.388268	-0.351511	0.091109
57	6	0	13.700837	0.842702	-0.009515
58	8	0	-14.343410	1.348391	-0.482807
59	8	0	-13.908563	-3.184811	0.056744
60	8	0	13.973395	-2.620548	0.276135
61	8	0	14.182956	1.947781	-0.066200
62	6	0	-15.877763	-1.100950	-0.331106
63	6	0	15.840881	-0.412537	0.162132
64	6	0	-5.014341	4.124507	0.423336
65	6	0	4.299484	-4.013869	-0.352605
66	1	0	0.044348	-2.111626	-0.077014
67	1	0	-2.005996	-1.179574	0.009720
68	1	0	-2.457601	3.550553	0.278703
69	1	0	1.849211	-3.042146	-0.203734
70	1	0	-9.254201	2.771783	-0.290434
71	1	0	-6.974280	3.149476	-0.214529
72	1	0	-5.103053	-1.438709	0.578909
73	1	0	-6.251003	-3.431446	0.699193
74	1	0	-8.680191	-3.563951	0.491206
75	1	0	6.421960	-3.340836	0.111298
76	1	0	8.730475	-3.294245	0.146271
77	1	0	9.058907	3.085779	-0.360603
78	1	0	6.631777	3.313417	-0.495864
79	1	0	5.212262	1.507929	-0.419496
80	1	0	-11.085122	-3.085272	0.243762
81	1	0	-11.554969	1.826820	-0.340172
82	1	0	11.371972	2.258578	-0.200403
83	1	0	11.143395	-2.691140	0.171487
84	1	0	-16.164444	-2.082448	-0.770525
85	1	0	-16.316412	-1.023995	0.689649
86	1	0	-16.334723	-0.317860	-0.976669
87	1	0	16.230000	-1.358325	-0.276853
88	1	0	16.311310	0.419702	-0.407773
89	1	0	16.169189	-0.349146	1.224403
90	1	0	-5.780122	4.141626	1.231013
91	1	0	-4.256227	4.865863	0.762147
92	1	0	-5.408508	4.527404	-0.536922
93	1	0	4.997219	-4.171620	-1.205616
94	1	0	4.693993	-4.437919	0.598346
95	1	0	3.419992	-4.646710	-0.607278

Optimized structure of **BTz-T-FA-5**

Center Number	Atomic Number	Atomic Type	Coordinates (Angstroms) X	Y	Z
1	6	0	0.208143	1.459625	0.064859
2	6	0	-1.040369	0.976874	-0.025102
3	6	0	-1.327003	-0.328511	-0.216470
4	6	0	-0.224916	-1.107586	-0.302137
5	6	0	1.040527	-0.622853	-0.208694
6	6	0	1.310942	0.690492	-0.019741
7	7	0	-0.261926	-2.363168	-0.467605
8	16	0	1.275115	-3.081637	-0.537137
9	7	0	1.923804	-1.528039	-0.314271
10	6	0	-2.612233	-0.760180	-0.301621
11	6	0	2.540139	1.254535	0.087679
12	16	0	-3.791565	0.110554	-0.189869
13	6	0	-4.436874	-2.027226	-0.521882
14	6	0	-3.098410	-1.983158	-0.556359
15	6	0	-4.931932	-0.821132	-0.180027
16	16	0	3.832007	0.574827	0.029898
17	6	0	4.165076	2.766480	0.349011
18	6	0	2.844077	2.543118	0.285862
19	6	0	4.857257	1.615551	0.178558
20	6	0	-6.220068	-0.411308	-0.044360
21	6	0	6.195336	1.342388	0.156473
22	6	0	-7.247619	-1.218554	0.321885
23	6	0	-8.496223	-0.738728	0.252620
24	6	0	-8.812706	0.504207	-0.112047
25	6	0	-7.817412	1.345830	-0.395852
26	6	0	-6.557870	0.873644	-0.340894
27	6	0	-7.193507	-2.438570	0.893825
28	6	0	-8.294973	-3.146014	1.213539
29	6	0	-9.531089	-2.650950	1.030439
30	6	0	-9.615853	-1.399524	0.567602
31	6	0	7.052409	2.395275	0.140183
32	6	0	8.394246	2.342969	0.110671
33	6	0	8.954749	1.136517	0.091302
34	6	0	8.148176	0.073167	0.104447
35	6	0	6.802758	0.113631	0.138370
36	6	0	8.928281	-1.016832	0.083897
37	6	0	8.373559	-2.230915	0.101468

38	6	0	7.034216	-2.265830	0.152354
39	6	0	6.292073	-1.140828	0.175111
40	6	0	-10.148454	0.613193	-0.072400
41	6	0	-10.646316	-0.569394	0.345596
42	6	0	-11.964029	-0.767793	0.500873
43	6	0	-12.753116	0.276618	0.209192
44	6	0	-12.257009	1.452631	-0.206619
45	6	0	-10.940270	1.657866	-0.357197
46	6	0	12.500123	0.694344	0.000873
47	6	0	12.484744	-0.647927	-0.003332
48	6	0	11.341249	-1.348265	0.022417
49	6	0	10.211100	-0.625537	0.051302
50	6	0	10.228376	0.721207	0.055718
51	6	0	11.372939	1.420266	0.031031
52	6	0	-14.104499	0.311120	0.291202
53	7	0	-14.361852	1.602344	-0.127316
54	6	0	-13.243002	2.353542	-0.431475
55	6	0	13.766145	1.174643	-0.024302
56	7	0	14.493476	0.000644	-0.059869
57	6	0	13.739591	-1.156722	-0.031762
58	8	0	-13.225920	3.504277	-0.794881
59	8	0	-14.920016	-0.512968	0.626683
60	8	0	14.210222	2.296864	-0.018831
61	8	0	14.158903	-2.288502	-0.033553
62	6	0	-15.710174	2.146651	-0.196410
63	6	0	15.948954	-0.016280	-0.077087
64	6	0	-5.143170	-3.277569	-0.989877
65	6	0	4.625427	4.176112	0.643761
66	1	0	0.269380	2.548418	0.217760
67	1	0	-1.829901	1.742642	0.067171
68	1	0	-2.535859	-2.892297	-0.794038
69	1	0	2.124347	3.363866	0.402007
70	1	0	-8.026913	2.387566	-0.687764
71	1	0	-5.807926	1.624286	-0.644562
72	1	0	-6.227188	-2.881488	1.173374
73	1	0	-8.183833	-4.142580	1.674845
74	1	0	-10.426029	-3.225198	1.315651
75	1	0	6.706806	3.430404	0.100780
76	1	0	9.010664	3.256085	0.082995
77	1	0	8.974754	-3.152746	0.088318
78	1	0	6.537216	-3.251749	0.184908
79	1	0	5.222047	-1.367402	0.239703
80	1	0	-12.372879	-1.728145	0.848952
81	1	0	-10.533960	2.623847	-0.692421
82	1	0	11.332743	-2.448364	0.021168
83	1	0	11.386823	2.520350	0.036906
84	1	0	-16.456044	1.356349	-0.436712
85	1	0	-15.980396	2.608610	0.780236
86	1	0	-15.796581	2.918636	-0.993450
87	1	0	16.360172	0.876383	-0.599243
88	1	0	16.337763	-0.031273	0.966306
89	1	0	16.338404	-0.908182	-0.617094
90	1	0	-6.182658	-3.089955	-1.335522
91	1	0	-4.625846	-3.679676	-1.891028
92	1	0	-5.110700	-4.091323	-0.231644
93	1	0	5.331373	4.210035	1.503850
94	1	0	5.043278	4.668522	-0.263206
95	1	0	3.782904	4.828768	0.964738

Optimized structure of **BTz-T-FA-6**

Center Number	Atomic Number	Atomic Type	Coordinates (Angstroms) X	Y	Z
1	6	0	-0.075238	-0.557483	-0.137171
2	6	0	1.171006	-0.061227	-0.138169
3	6	0	1.454067	1.252340	-0.007298
4	6	0	0.351239	2.022813	0.129060
5	6	0	-0.911981	1.523204	0.133639
6	6	0	-1.179513	0.202612	-0.001835
7	7	0	0.386066	3.281243	0.272072
8	16	0	-1.151394	3.986241	0.423292
9	7	0	-1.796321	2.422038	0.277045
10	6	0	2.737687	1.695946	-0.016008
11	6	0	-2.405412	-0.379452	-0.003406
12	16	0	3.915674	0.835145	-0.150968
13	6	0	4.574263	2.967851	0.037283
14	6	0	3.233844	2.936212	0.062420
15	6	0	5.084487	1.717244	-0.041208
16	16	0	-3.701925	0.287979	0.137106
17	6	0	-4.006727	-1.928532	-0.093042
18	6	0	-2.694307	-1.676168	-0.180996
19	6	0	-4.692635	-0.803428	0.205802
20	6	0	6.369216	1.255743	-0.057840
21	6	0	-6.038799	-0.614460	0.381379
22	6	0	6.802491	-0.014056	-0.334584
23	6	0	8.123916	-0.260996	-0.263296
24	6	0	9.062726	0.634745	0.049020
25	6	0	8.672958	1.879012	0.314027
26	6	0	7.355904	2.135274	0.252781
27	6	0	6.129654	-1.126679	-0.714562
28	6	0	6.709411	-2.320298	-0.951812
29	6	0	8.033343	-2.500823	-0.839323
30	6	0	8.745430	-1.426645	-0.491127
31	6	0	-6.500857	0.633230	0.090985
32	6	0	-7.791107	0.973474	-0.022175
33	6	0	-8.661145	-0.017240	0.140770
34	6	0	-8.201250	-1.218602	0.512309
35	6	0	-6.932069	-1.617119	0.758156
36	6	0	-9.290153	-1.977239	0.599152
37	6	0	-9.028022	-3.231003	0.920479
38	6	0	-7.750824	-3.464129	1.245889
39	6	0	-6.571153	-2.829392	1.410215
40	6	0	10.261945	0.037203	0.029136
41	6	0	10.062966	-1.251631	-0.308436
42	6	0	11.079932	-2.120179	-0.416201
43	6	0	12.301608	-1.627399	-0.164764
44	6	0	12.498131	-0.342828	0.171685
45	6	0	11.484828	0.529168	0.277880
46	6	0	-12.157944	0.543635	-0.380243
47	6	0	-12.552596	-0.709620	-0.102687
48	6	0	-11.683499	-1.671692	0.240435
49	6	0	-10.397881	-1.295702	0.290256
50	6	0	-9.996427	-0.043545	0.010146
51	6	0	-10.869565	0.914989	-0.334709
52	6	0	13.470335	-2.310362	-0.211363
53	7	0	14.368644	-1.321764	0.141494
54	6	0	13.811261	-0.079063	0.372558
55	6	0	-13.210781	1.332487	-0.702232
56	7	0	-14.258729	0.441371	-0.573774
57	6	0	-13.895169	-0.843347	-0.219395
58	8	0	14.401314	0.930687	0.670842
59	8	0	13.731825	-3.458212	-0.477323
60	8	0	-13.289575	2.493916	-1.021225
61	8	0	-14.634076	-1.785983	-0.071098
62	6	0	15.802561	-1.561280	0.217657
63	6	0	-15.639829	0.817373	-0.838912
64	6	0	5.248835	4.321184	0.040264

65	6	0	-4.510233	-3.311553	-0.432504
66	1	0	-0.132233	-1.651407	-0.249328
67	1	0	1.962011	-0.822095	-0.256238
68	1	0	2.664108	3.868273	0.136708
69	1	0	-1.982825	-2.485839	-0.389269
70	1	0	9.402637	2.654181	0.598669
71	1	0	7.147428	3.164878	0.553655
72	1	0	5.047143	-1.160188	-0.881276
73	1	0	6.087729	-3.180451	-1.257825
74	1	0	8.501463	-3.476812	-1.038960
75	1	0	-5.850032	1.482625	-0.177330
76	1	0	-8.109315	1.986505	-0.314882
77	1	0	-9.810568	-4.004622	0.957287
78	1	0	-7.602931	-4.525931	1.533976
79	1	0	-5.721770	-2.181140	1.492507
80	1	0	10.923048	-3.173293	-0.693291
81	1	0	11.647133	1.582306	0.551906
82	1	0	-12.001171	-2.699501	0.470595
83	1	0	-10.550390	1.941580	-0.568060
84	1	0	16.023537	-2.605203	0.533965
85	1	0	16.267735	-1.382721	-0.778278
86	1	0	16.287785	-0.893216	0.963812
87	1	0	-15.826439	1.884417	-0.583343
88	1	0	-15.872565	0.661166	-1.916717
89	1	0	-16.349807	0.215323	-0.228881
90	1	0	6.022439	4.401783	-0.755989
91	1	0	4.533329	5.138723	-0.202191
92	1	0	5.654531	4.574732	1.045666
93	1	0	-5.528065	-3.293836	-0.880620
94	1	0	-3.870991	-3.774433	-1.218250
95	1	0	-4.467730	-3.994544	0.445153

Optimized structure of **BTz-T-FA-7**

Center Number	Atomic Number	Atomic Type	Coordinates (Angstroms) X	Y	Z
1	6	0	-0.224911	0.962284	-0.264635
2	6	0	1.049375	0.560136	-0.145362
3	6	0	1.410205	-0.699278	0.180855
4	6	0	0.353546	-1.522560	0.367124
5	6	0	-0.937887	-1.118791	0.246639
6	6	0	-1.283414	0.150156	-0.076356
7	7	0	0.461907	-2.748965	0.665877
8	16	0	-1.031919	-3.537158	0.840737
9	7	0	-1.768084	-2.053356	0.466067
10	6	0	2.718274	-1.048262	0.294412
11	6	0	-2.542968	0.636798	-0.213024
12	16	0	3.846291	-0.129947	0.080203
13	6	0	4.612019	-2.181303	0.633881
14	6	0	3.273249	-2.209890	0.668389
15	6	0	5.038775	-0.989082	0.172014
16	16	0	-3.797279	-0.104768	-0.050723
17	6	0	-4.234180	2.044032	-0.592635
18	6	0	-2.905225	1.875498	-0.578665
19	6	0	-4.848278	0.920152	-0.171042
20	6	0	6.303133	-0.520872	0.005024
21	6	0	-6.170703	0.632039	-0.049702
22	6	0	7.380058	-1.299871	-0.268670
23	6	0	8.598718	-0.745397	-0.231302
24	6	0	8.839166	0.543198	0.013489
25	6	0	7.793657	1.350535	0.199038
26	6	0	6.563219	0.804408	0.175670
27	6	0	7.403442	-2.571082	-0.717733
28	6	0	8.547964	-3.242794	-0.951757
29	6	0	9.751354	-2.662974	-0.802074
30	6	0	9.758695	-1.369644	-0.463762
31	6	0	-6.605097	-0.644009	-0.241533
32	6	0	-7.896540	-1.018591	-0.303163
33	6	0	-8.827248	-0.077858	-0.137278
34	6	0	-8.421123	1.165136	0.123895
35	6	0	-7.139861	1.548979	0.199027
36	6	0	-9.492800	1.938323	0.331486
37	6	0	-9.320705	3.217498	0.680213
38	6	0	-8.054683	3.628808	0.866563
39	6	0	-7.005104	2.809618	0.658204
40	6	0	10.167659	0.722606	-0.016764
41	6	0	10.737617	-0.465170	-0.308904
42	6	0	12.067276	-0.603845	-0.422402
43	6	0	12.792391	0.506111	-0.220921
44	6	0	12.224581	1.687410	0.069613
45	6	0	10.895707	1.832543	0.177219
46	6	0	-12.332202	-0.759676	-0.278695
47	6	0	-12.744035	0.485803	0.006733
48	6	0	-11.882170	1.489051	0.229306
49	6	0	-10.580577	1.174513	0.146395
50	6	0	-10.167973	-0.077722	-0.140740
51	6	0	-11.032525	-1.079706	-0.359813
52	6	0	14.141541	0.607651	-0.282342
53	7	0	14.319653	1.945327	0.012953
54	6	0	13.155526	2.659000	0.222807
55	6	0	-13.380287	-1.597520	-0.462036
56	7	0	-14.443359	-0.742571	-0.244148
57	6	0	-14.095808	0.565146	0.034048
58	8	0	13.068761	3.836732	0.472218
59	8	0	15.007459	-0.198391	-0.521332
60	8	0	-13.445450	-2.770517	-0.738662
61	8	0	-14.852251	1.483446	0.237247
62	6	0	15.635032	2.567626	0.052871
63	6	0	-15.828664	-1.177916	-0.346519
64	6	0	5.385907	-3.334479	1.228141
65	6	0	-4.806260	3.308634	-1.187514
66	1	0	-0.346853	2.024770	-0.526877
67	1	0	1.794350	1.353107	-0.331211
68	1	0	2.763297	-3.119606	1.002197
69	1	0	-2.237840	2.697787	-0.868344
70	1	0	7.939754	2.425864	0.390123
71	1	0	5.767422	1.536732	0.395436
72	1	0	6.467784	-3.093396	-0.962942
73	1	0	8.500257	-4.284699	-1.312927
74	1	0	10.681678	-3.210983	-1.016533
75	1	0	-5.912472	-1.478683	-0.445592
76	1	0	-8.181579	-2.063326	-0.506725
77	1	0	-10.174314	3.884615	0.875462
78	1	0	-7.877310	4.652638	1.238816
79	1	0	-6.015795	3.199474	0.935611
80	1	0	12.534709	-1.569253	-0.667481
81	1	0	10.430674	2.802104	0.410304
82	1	0	-12.222556	2.507649	0.468543
83	1	0	-10.695569	-2.101478	-0.590257
84	1	0	16.416131	1.846780	0.383307
85	1	0	15.903186	2.946723	-0.959306
86	1	0	15.661472	3.416880	0.771715
87	1	0	-15.942891	-2.243570	-0.046467
88	1	0	-16.491927	-0.586036	0.323300
89	1	0	-16.183620	-1.061331	-1.395574
90	1	0	5.409868	-4.219929	0.554797

91	1	0	6.408607	-3.051482	1.558659
92	1	0	4.884368	-3.675737	2.162674
93	1	0	-5.845607	3.184757	-1.561272
94	1	0	-4.725777	4.176075	-0.495307
95	1	0	-4.223048	3.587902	-2.094850

Optimized structure of **BTz-T-FA-8**

Center	Atomic	Atomic	Coordinates (Angstroms)		
Number	Number	Type	X	Y	Z
1	6	0	0.670189	1.958837	-0.033660
2	6	0	-0.669965	1.958779	0.033777
3	6	0	-1.418126	3.081754	0.074367
4	6	0	-0.678068	4.212571	0.036155
5	6	0	0.678210	4.212549	-0.035080
6	6	0	1.418269	3.081864	-0.073821
7	7	0	-1.170766	5.379662	0.058959
8	16	0	0.000005	6.608312	0.000868
9	7	0	1.170818	5.379659	-0.057454
10	6	0	-2.772935	3.020678	0.146711
11	6	0	2.773030	3.020840	-0.146267
12	16	0	-3.553104	1.780711	0.176268
13	6	0	-4.944408	3.533991	0.272305
14	6	0	-3.685897	3.995248	0.231859
15	6	0	-4.965341	2.183464	0.193654
16	16	0	3.553271	1.780805	-0.176455
17	6	0	4.944433	3.534227	-0.271848
18	6	0	3.685980	3.995371	-0.231190
19	6	0	4.965511	2.183652	-0.193731
20	6	0	-5.996580	1.289388	0.165729
21	6	0	5.996766	1.289527	-0.166428
22	6	0	-5.931061	-0.073533	0.287327
23	6	0	-7.078682	-0.772052	0.202068
24	6	0	-8.291571	-0.247552	0.014511
25	6	0	-8.387788	1.074631	-0.099682
26	6	0	-7.248025	1.781141	-0.022673
27	6	0	-4.884474	-0.901840	0.518332
28	6	0	-4.985116	-2.242766	0.613181
29	6	0	-6.161215	-2.876320	0.495742
30	6	0	-7.228808	-2.101040	0.291805
31	6	0	7.248297	1.781175	0.021487
32	6	0	8.388114	1.074684	0.097478
33	6	0	8.291871	-0.247435	-0.017049
34	6	0	7.078874	-0.771836	-0.203821
35	6	0	5.931119	-0.073355	-0.288163
36	6	0	7.228989	-2.100850	-0.293691
37	6	0	6.161309	-2.876171	-0.496749
38	6	0	4.985037	-2.242725	-0.613372
39	6	0	4.884477	-0.901749	-0.518388
40	6	0	-9.195020	-1.236370	-0.024609
41	6	0	-8.530067	-2.395049	0.148680
42	6	0	-9.162397	-3.578432	0.161471
43	6	0	-10.491685	-3.535576	-0.011528
44	6	0	-11.152336	-2.379791	-0.184489
45	6	0	-10.524821	-1.194814	-0.195211
46	6	0	11.152828	-2.379699	0.179297
47	6	0	10.492134	-3.535416	0.006806
48	6	0	9.162435	-3.578360	-0.163596
49	6	0	8.530283	-2.394841	-0.151673
50	6	0	9.195371	-1.236219	0.021346
51	6	0	10.525067	-1.194774	0.192348
52	6	0	-11.335347	-4.594920	-0.034470
53	7	0	-12.545583	-3.964816	-0.251210
54	6	0	-12.482259	-2.587336	-0.334495
55	6	0	12.482240	-2.587555	0.333294
56	7	0	12.549151	-3.962773	0.221197
57	6	0	11.335091	-4.595174	0.033817
58	8	0	-13.409476	-1.832382	-0.498544
59	8	0	-11.154571	-5.781598	0.091459
60	8	0	13.406853	-1.834411	0.519248
61	8	0	11.151310	-5.783502	-0.069461
62	6	0	-13.801659	-4.695470	-0.337555
63	6	0	13.801336	-4.695525	0.339499
64	6	0	-6.059318	4.539411	0.453280
65	6	0	6.059253	4.539808	-0.452498
66	1	0	1.124393	0.952999	-0.057504
67	1	0	-1.124177	0.952916	0.057096
68	1	0	-3.495399	5.072485	0.274977
69	1	0	3.495411	5.072736	-0.273680
70	1	0	-9.360882	1.560128	-0.278026
71	1	0	-7.438337	2.842907	-0.197665
72	1	0	-3.854211	-0.558965	0.666246
73	1	0	-4.080947	-2.848148	0.802987
74	1	0	-6.237589	-3.971116	0.579247
75	1	0	7.438681	2.842910	0.196606
76	1	0	9.361311	1.560088	0.275335
77	1	0	6.237492	-3.970977	-0.580183
78	1	0	4.080661	-2.847943	-0.802396
79	1	0	3.854160	-0.559022	-0.665394
80	1	0	-8.624616	-4.527568	0.304558
81	1	0	-11.067420	-0.247984	-0.335128
82	1	0	8.624285	-4.527726	-0.303910
83	1	0	11.067409	-0.248203	0.334588
84	1	0	-13.653149	-5.708685	-0.773639
85	1	0	-14.244850	-4.804223	0.678265
86	1	0	-14.533086	-4.171371	-0.992581
87	1	0	14.660381	-4.095560	-0.035575
88	1	0	13.985824	-4.956502	1.406382
89	1	0	13.782382	-5.633046	-0.259945
90	1	0	-6.766737	4.236032	1.257424
91	1	0	-5.673413	5.524588	0.799137
92	1	0	-6.582023	4.749270	-0.507138
93	1	0	6.766424	4.237187	-1.257151
94	1	0	5.673304	5.525333	-0.797329
95	1	0	6.582439	4.748951	0.507929

Optimized structure of **BTz-T-FA-9**

Center	Atomic	Atomic	Coordinates (Angstroms)		
Number	Number	Type	X	Y	Z
1	6	0	-0.488267	1.561908	-0.174500
2	6	0	0.851790	1.629949	-0.187520
3	6	0	1.542968	2.789339	-0.209649
4	6	0	0.745376	3.880892	-0.208060
5	6	0	-0.610953	3.811798	-0.195276
6	6	0	-1.293985	2.645020	-0.184337
7	7	0	1.178693	5.071479	-0.214148
8	16	0	-0.054222	6.239231	-0.203907
9	7	0	-1.162468	4.952563	-0.187813
10	6	0	2.900899	2.797211	-0.227209
11	6	0	-2.646355	2.516704	-0.178274
12	16	0	3.743075	1.598163	-0.222347

13	6	0	5.046990	3.419578	-0.269455
14	6	0	3.766193	3.816593	-0.277641
15	6	0	5.133016	2.071587	-0.191946
16	16	0	-3.361662	1.232504	-0.158611
17	6	0	-4.838456	2.940439	-0.191600
18	6	0	-3.602491	3.453370	-0.252944
19	6	0	-4.778690	1.605851	-0.012999
20	6	0	6.206062	1.229841	-0.131709
21	6	0	-5.779731	0.691336	0.073535
22	6	0	6.212615	-0.133956	-0.260219
23	6	0	7.390718	-0.774600	-0.142485
24	6	0	8.569289	-0.191107	0.085250
25	6	0	8.595298	1.133395	0.209287
26	6	0	7.424587	1.782496	0.099145
27	6	0	5.216810	-1.012496	-0.527366
28	6	0	5.387709	-2.346123	-0.625444
29	6	0	6.590131	-2.920429	-0.474987
30	6	0	7.610356	-2.093731	-0.234353
31	6	0	-5.586362	-0.571713	-0.396332
32	6	0	-6.548935	-1.505724	-0.512536
33	6	0	-7.780780	-1.186154	-0.112975
34	6	0	-7.972100	0.020944	0.420156
35	6	0	-7.021735	0.955588	0.552096
36	6	0	-9.242787	0.122871	0.825330
37	6	0	-9.643128	1.231147	1.456921
38	6	0	-8.703478	2.159812	1.704383
39	6	0	-7.430958	2.009366	1.287203
40	6	0	9.519897	-1.133581	0.146530
41	6	0	8.919878	-2.322848	-0.053544
42	6	0	9.611308	-3.472843	-0.054675
43	6	0	10.931074	-3.364403	0.158023
44	6	0	11.526963	-2.178133	0.357785
45	6	0	10.840217	-1.026353	0.357260
46	6	0	-10.550540	-3.433619	-0.337155
47	6	0	-11.453939	-2.624341	0.238248
48	6	0	-11.137429	-1.395199	0.671411
49	6	0	-9.862167	-1.019533	0.491134
50	6	0	-8.954738	-1.833575	-0.087481
51	6	0	-9.273817	-3.064085	-0.515896
52	6	0	11.826132	-4.379967	0.198824
53	7	0	12.996067	-3.691426	0.455407
54	6	0	12.860653	-2.319630	0.545933
55	6	0	-11.102599	-4.618737	-0.690570
56	7	0	-12.403569	-4.443346	-0.259755
57	6	0	-12.671377	-3.213397	0.309330
58	8	0	13.743340	-1.520217	0.742255
59	8	0	11.709528	-5.573213	0.059725
60	8	0	-10.649721	-5.598027	-1.231354
61	8	0	-13.734846	-2.833470	0.735284
62	6	0	14.284556	-4.358280	0.574070
63	6	0	-13.428396	-5.462046	-0.435791
64	6	0	6.115743	4.480317	-0.408162
65	6	0	-6.019816	3.837610	-0.476760
66	1	0	-0.890743	0.534233	-0.160173
67	1	0	1.357136	0.648489	-0.181742
68	1	0	3.523078	4.882864	-0.329413
69	1	0	-3.473607	4.533931	-0.375877
70	1	0	9.536805	1.665914	0.420187
71	1	0	7.555594	2.851494	0.284290
72	1	0	4.175511	-0.721355	-0.705549
73	1	0	4.521355	-2.994849	-0.846054
74	1	0	6.724145	-4.009434	-0.561646
75	1	0	-4.616797	-0.906049	-0.803869
76	1	0	-6.336892	-2.496300	-0.946066
77	1	0	-10.675552	1.350186	1.820212
78	1	0	-8.977241	3.048842	2.298546
79	1	0	-6.710744	2.769322	1.622011
80	1	0	9.126760	-4.446739	-0.219947
81	1	0	11.329843	-0.054428	0.518880
82	1	0	-11.878720	-0.735744	1.147087
83	1	0	-8.533231	-3.728997	-0.985123
84	1	0	14.174962	-5.382860	0.994658
85	1	0	14.764302	-4.432182	-0.428328
86	1	0	14.967300	-3.805326	1.257297
87	1	0	-12.994867	-6.486254	-0.395550
88	1	0	-14.196195	-5.409593	0.368140
89	1	0	-13.931038	-5.324469	-1.420096
90	1	0	6.864533	4.216843	-1.188561
91	1	0	5.693315	5.447107	-0.762995
92	1	0	6.594083	4.710620	0.570495
93	1	0	-6.913202	3.283715	-0.837744
94	1	0	-6.272323	4.492280	0.386825
95	1	0	-5.769414	4.524763	-1.317196

Optimized structure of **BTz-T-FA-10**

Center Number	Atomic Number	Atomic Type	Coordinates (Angstroms) X	Y	Z
1	6	0	0.670623	-1.212714	0.017355
2	6	0	-0.670725	-1.212850	-0.017300
3	6	0	-1.419981	-2.335605	-0.040985
4	6	0	-0.678760	-3.466143	-0.018474
5	6	0	0.678880	-3.466011	0.019620
6	6	0	1.419987	-2.335404	0.041496
7	7	0	-1.171720	-4.633275	-0.024620
8	16	0	0.000206	-5.862125	0.001262
9	7	0	1.171910	-4.633126	0.026393
10	6	0	-2.776453	-2.276138	-0.082190
11	6	0	2.776480	-2.275768	0.082574
12	16	0	-3.556395	-1.029976	-0.095925
13	6	0	-4.942540	-2.810725	-0.164758
14	6	0	-3.680894	-3.260439	-0.182740
15	6	0	-4.956461	-1.474269	0.009641
16	16	0	3.556209	-1.029480	0.095708
17	6	0	4.942599	-2.810002	0.165098
18	6	0	3.681006	-3.260006	0.183480
19	6	0	4.956298	-1.473604	-0.009940
20	6	0	-6.004186	-0.611094	0.064323
21	6	0	6.003874	-0.610343	-0.065129
22	6	0	-7.244922	-0.936742	0.507336
23	6	0	-8.236304	-0.050494	0.347283
24	6	0	-8.090005	1.165115	-0.181019
25	6	0	-6.864622	1.545342	-0.545988
26	6	0	-5.860698	0.660042	-0.401605
27	6	0	-7.622321	-2.009537	1.231901
28	6	0	-8.897233	-2.223184	1.612695
29	6	0	-9.874277	-1.342056	1.337768
30	6	0	-9.511393	-0.215123	0.716747
31	6	0	5.860375	0.660870	0.400272
32	6	0	6.864219	1.546405	0.544013
33	6	0	8.089499	1.166119	0.178644
34	6	0	8.235881	-0.049620	-0.349170
35	6	0	7.244467	-0.936208	-0.508383
36	6	0	9.510877	-0.214396	-0.718810
37	6	0	9.873815	-1.341709	-1.338987
38	6	0	8.896802	-2.223116	-1.613260

39	6	0	7.621976	-2.009356	-1.232348
40	6	0	-9.294448	1.754023	-0.188501
41	6	0	-10.176659	0.895736	0.364886
42	6	0	-11.473033	1.208871	0.510789
43	6	0	-11.837465	2.421145	0.067982
44	6	0	-10.959193	3.274451	-0.482222
45	6	0	-9.661284	2.968601	-0.624311
46	6	0	10.958430	3.276170	0.477543
47	6	0	11.836671	2.422643	-0.072427
48	6	0	11.472554	1.209565	-0.513037
49	6	0	10.175993	0.896790	-0.367739
50	6	0	9.293974	1.755235	0.185429
51	6	0	9.660909	2.969602	0.621748
52	6	0	-13.082678	2.951926	0.110219
53	7	0	-12.865144	4.183490	-0.476592
54	6	0	-11.557537	4.433823	-0.845556
55	6	0	11.557689	4.433831	0.844726
56	7	0	12.859696	4.194728	0.449342
57	6	0	13.082790	2.951771	-0.110831
58	8	0	-11.133827	5.443720	-1.352802
59	8	0	-14.133338	2.528865	0.527338
60	8	0	11.138405	5.435217	1.372288
61	8	0	14.137726	2.519660	-0.507275
62	6	0	-13.926824	5.164383	-0.649473
63	6	0	13.927406	5.163293	0.651653
64	6	0	-6.067114	-3.767878	-0.482087
65	6	0	6.067387	-3.766888	0.482762
66	1	0	1.124792	-0.206760	0.029577
67	1	0	-1.125017	-0.206964	-0.030016
68	1	0	-3.493525	-4.333731	-0.294050
69	1	0	3.493744	-4.333257	0.295189
70	1	0	-6.689662	2.545628	-0.973933
71	1	0	-4.897674	1.042378	-0.781389
72	1	0	-6.875418	-2.732789	1.588514
73	1	0	-9.143372	-3.124769	2.199915
74	1	0	-10.909410	-1.511985	1.671989
75	1	0	4.897378	1.043248	0.780360
76	1	0	6.689367	2.546759	0.971648
77	1	0	10.908890	-1.511886	-1.673199
78	1	0	9.142923	-3.125066	-2.199886
79	1	0	6.875016	-2.732832	-1.588452
80	1	0	-12.193523	0.514349	0.968193
81	1	0	-8.940874	3.670608	-1.070204
82	1	0	12.193526	0.513954	-0.968048
83	1	0	8.940988	3.670817	1.069769
84	1	0	-14.912345	4.672669	-0.810002
85	1	0	-13.992628	5.811475	0.254410
86	1	0	-13.744337	5.808889	-1.538316
87	1	0	13.545347	6.207106	0.595662
88	1	0	14.394424	5.006108	1.650329
89	1	0	14.714491	5.070270	-0.129732
90	1	0	-6.310960	-4.433966	0.375280
91	1	0	-6.976756	-3.261398	-0.870997
92	1	0	-5.756340	-4.441959	-1.312933
93	1	0	6.311166	-4.433425	-0.374230
94	1	0	6.976963	-3.260047	0.871336
95	1	0	5.756854	-4.440535	1.314162

Optimized structure of **BTz-TT-FA-1**

Center Number	Atomic Number	Atomic Type	Coordinates (Angstroms) X	Y	Z
1	6	0	-0.670793	-1.146165	-0.012744
2	6	0	0.670781	-1.146141	0.012166
3	6	0	1.425857	-0.026744	0.025803
4	6	0	0.679269	1.101147	0.011858
5	6	0	-0.679403	1.101157	-0.012105
6	6	0	-1.425898	-0.026756	-0.026165
7	7	0	1.170028	2.269070	0.020797
8	16	0	-0.000077	3.499507	-0.000028
9	7	0	-1.170196	2.269138	-0.020740
10	6	0	2.785309	-0.087356	0.050173
11	6	0	-2.785292	-0.087345	-0.050619
12	16	0	3.534697	-1.373252	0.069730
13	6	0	4.902669	-0.870880	0.092405
14	6	0	4.922192	0.450183	0.086386
15	6	0	3.678579	0.928064	0.063953
16	6	0	6.152057	-1.349474	0.106222
17	6	0	7.043067	-0.316454	0.084607
18	16	0	6.280303	0.956176	0.121287
19	16	0	-3.534617	-1.373354	-0.070450
20	6	0	-4.902683	-0.871024	-0.093253
21	6	0	-4.922228	0.450018	-0.087068
22	6	0	-3.678605	0.928039	-0.064357
23	6	0	-6.151984	-1.349732	-0.107449
24	6	0	-7.043067	-0.316748	-0.085695
25	16	0	-6.280319	0.955927	-0.122197
26	6	0	8.408401	-0.337582	0.072755
27	6	0	-8.408386	-0.337920	-0.074038
28	6	0	9.270594	0.689734	0.347818
29	6	0	10.590834	0.452883	0.237814
30	6	0	11.141179	-0.712265	-0.110012
31	6	0	10.328031	-1.732178	-0.374107
32	6	0	9.007639	-1.505128	-0.276102
33	6	0	9.044164	1.954445	0.775866
34	6	0	10.015097	2.860539	1.006853
35	6	0	11.313484	2.565824	0.845415
36	6	0	11.590262	1.317718	0.460331
37	6	0	-9.007589	-1.505680	0.274415
38	6	0	-10.327989	-1.732730	0.372178
39	6	0	-11.141263	-0.712939	0.108025
40	6	0	-10.590862	0.452318	-0.239297
41	6	0	-9.270675	0.689368	-0.348861
42	6	0	-11.590335	1.317186	-0.461533
43	6	0	-11.313669	2.565486	-0.845733
44	6	0	-10.015304	2.860472	-1.007046
45	6	0	-9.044291	1.954362	-0.776429
46	6	0	12.474525	-0.578428	-0.120897
47	6	0	12.754614	0.690262	0.235493
48	6	0	14.016052	1.138955	0.323261
49	6	0	14.976803	0.249397	0.032701
50	6	0	14.695926	-1.014392	-0.322588
51	6	0	13.436782	-1.467390	-0.409461
52	6	0	-14.696023	-1.015613	0.318977
53	6	0	-14.976950	0.248203	-0.035832
54	6	0	-14.016139	1.138348	-0.324318
55	6	0	-12.754695	0.689540	-0.237336
56	6	0	-12.474575	-0.579275	0.118576
57	6	0	-13.436782	-1.468117	0.407665
58	6	0	16.312612	0.471233	0.054598
59	7	0	16.792609	-0.764980	-0.332931
60	6	0	15.824575	-1.723479	-0.562120
61	6	0	-15.824592	-1.723629	0.562488

62	7	0	-16.793485	-0.773026	0.305860
63	6	0	-16.312759	0.471269	-0.053485
64	8	0	16.010015	-2.870994	-0.887172
65	8	0	16.971088	1.445755	0.325713
66	8	0	-16.009546	-2.865016	0.908529
67	8	0	-16.970270	1.452251	-0.302751
68	6	0	18.215849	-1.049537	-0.444805
69	6	0	-18.215461	-1.049368	0.449301
70	6	0	6.369057	-2.835888	0.280930
71	6	0	-6.368822	-2.836025	-0.282480
72	1	0	-1.117334	-2.155476	-0.021121
73	1	0	1.117289	-2.155433	0.020099
74	1	0	3.506129	2.009010	0.060978
75	1	0	-3.506337	2.008997	-0.061155
76	1	0	10.726423	-2.707818	-0.695851
77	1	0	8.433316	-2.377226	-0.604356
78	1	0	8.047548	2.353366	0.997283
79	1	0	9.747050	3.874122	1.354343
80	1	0	12.102101	3.307577	1.043811
81	1	0	-8.433250	-2.377885	0.602490
82	1	0	-10.726380	-2.708594	0.693669
83	1	0	-12.102221	3.307336	-1.043817
84	1	0	-9.747306	3.874193	-1.353993
85	1	0	-8.047580	2.353343	-0.997548
86	1	0	14.250103	2.173229	0.616404
87	1	0	13.207738	-2.504073	-0.698118
88	1	0	-14.250251	2.173280	-0.614923
89	1	0	-13.207673	-2.504238	0.698184
90	1	0	18.786818	-0.146717	-0.757448
91	1	0	18.608379	-1.399492	0.536893
92	1	0	18.415639	-1.833039	-1.209543
93	1	0	-18.448412	-2.112236	0.215259
94	1	0	-18.537739	-0.833786	1.493340
95	1	0	-18.822792	-0.429646	-0.247654
96	1	0	5.479462	-3.331353	0.729176
97	1	0	6.536030	-3.342851	-0.696256
98	1	0	7.181958	-3.059915	1.007286
99	1	0	-5.478905	-3.331363	-0.730301
100	1	0	-6.536121	-3.343263	0.694535
101	1	0	-7.181319	-3.060099	-1.009180

Optimized structure of **BTz-TT-FA-2**

Center Number	Atomic Number	Atomic Type	Coordinates (Angstroms) X	Y	Z
1	6	0	-0.595979	-0.780224	-0.224317
2	6	0	0.744556	-0.728565	-0.189882
3	6	0	1.450695	0.389609	0.083058
4	6	0	0.655827	1.457612	0.322610
5	6	0	-0.701531	1.405399	0.287441
6	6	0	-1.398824	0.280007	0.009900
7	7	0	1.095022	2.611985	0.604388
8	16	0	-0.127055	3.763708	0.856416
9	7	0	-1.242417	2.522194	0.543220
10	6	0	2.811718	0.383946	0.101600
11	6	0	-2.754436	0.170088	-0.041201
12	16	0	3.616587	-0.838641	-0.172026
13	6	0	4.957435	-0.291419	-0.016373
14	6	0	4.924360	0.998662	0.273204
15	6	0	3.658835	1.410967	0.342662
16	6	0	6.223623	-0.716480	-0.069453
17	6	0	7.053919	0.312847	0.246839
18	16	0	6.258068	1.562267	0.395148
19	16	0	-3.449518	-1.109119	-0.351744
20	6	0	-4.837371	-0.670133	-0.275591
21	6	0	-4.911808	0.615770	0.018324
22	6	0	-3.689243	1.127033	0.157713
23	6	0	-6.066135	-1.184084	-0.403288
24	6	0	-6.998562	-0.216876	-0.165216
25	16	0	-6.289277	1.063243	0.080932
26	6	0	8.413572	0.333186	0.259843
27	6	0	-8.361694	-0.294955	-0.167893
28	6	0	9.191037	-0.749666	0.516063
29	6	0	10.513973	-0.648447	0.332537
30	6	0	11.144502	0.463110	-0.047712
31	6	0	10.416013	1.566003	-0.226380
32	6	0	9.083783	1.475574	-0.055492
33	6	0	8.835087	-1.923278	1.077413
34	6	0	9.707445	-2.929898	1.282157
35	6	0	11.012932	-2.805639	0.987015
36	6	0	11.416762	-1.615346	0.531568
37	6	0	-8.912433	-1.532890	-0.076667
38	6	0	-10.222540	-1.826625	-0.032669
39	6	0	-11.076446	-0.806661	-0.078161
40	6	0	-10.573867	0.426136	-0.172689
41	6	0	-9.264553	0.732419	-0.226141
42	6	0	-11.606924	1.279001	-0.212756
43	6	0	-11.380777	2.589708	-0.328837
44	6	0	-10.095649	2.961963	-0.422295
45	6	0	-9.089162	2.066198	-0.381745
46	6	0	12.452242	0.180861	-0.135551
47	6	0	12.620160	-1.109887	0.220354
48	6	0	13.832102	-1.684869	0.245955
49	6	0	14.861046	-0.901517	-0.109143
50	6	0	14.692646	0.382466	-0.463045
51	6	0	13.484303	0.963560	-0.484306
52	6	0	-14.615643	-1.283680	0.062527
53	6	0	-14.946984	0.014435	-0.022001
54	6	0	-14.022889	0.981466	-0.120255
55	6	0	-12.744728	0.573440	-0.126081
56	6	0	-12.413827	-0.729855	-0.041747
57	6	0	-13.339637	-1.695878	0.054047
58	6	0	16.166107	-1.260314	-0.157098
59	7	0	16.748716	-0.082943	-0.584707
60	6	0	15.873489	0.969397	-0.772107
61	6	0	-15.714972	-2.070087	0.148684
62	7	0	-16.720242	-1.122806	0.123724
63	6	0	-16.290405	0.184409	0.002015
64	8	0	16.158616	2.089333	-1.120077
65	8	0	16.733975	-2.296329	0.089782
66	8	0	-15.854369	-3.266325	0.227778
67	8	0	-16.987059	1.168046	-0.060792
68	6	0	18.185156	0.052816	-0.777319
69	6	0	-18.130798	-1.479358	0.173641
70	6	0	6.542715	-2.062613	-0.678175
71	6	0	-6.224132	-2.604049	-0.898274
72	1	0	-0.998844	-1.778894	-0.465872
73	1	0	1.234412	-1.693184	-0.408455
74	1	0	3.437424	2.459232	0.566460
75	1	0	-3.562914	2.187388	0.397805
76	1	0	10.892630	2.512737	-0.527663
77	1	0	8.563262	2.422053	-0.281259
78	1	0	7.809398	-2.080727	1.441349
79	1	0	9.352515	-3.869326	1.740029
80	1	0	11.723642	-3.624249	1.178395
81	1	0	-8.302106	-2.430989	0.062814
82	1	0	-10.581091	-2.862980	0.075612

83	1	0	-12.198593	3.325172	-0.370938
84	1	0	-9.868869	4.035089	-0.551606
85	1	0	-8.109630	2.540070	-0.514123
86	1	0	13.973868	-2.734813	0.543127
87	1	0	13.347592	2.017515	-0.768903
88	1	0	-14.298287	2.044314	-0.191618
89	1	0	-13.069257	-2.760260	0.120702
90	1	0	18.645743	-0.909318	-1.095150
91	1	0	18.420375	0.795902	-1.571762
92	1	0	18.663279	0.381652	0.173271
93	1	0	-18.295085	-2.407505	0.765386
94	1	0	-18.734805	-0.680868	0.659902
95	1	0	-18.515345	-1.640341	-0.858973
96	1	0	5.874627	-2.250621	-1.549329
97	1	0	7.565143	-2.117246	-1.110029
98	1	0	6.363677	-2.900555	0.031692
99	1	0	-5.317366	-2.953186	-1.440331
100	1	0	-6.365945	-3.317773	-0.055571
101	1	0	-7.031740	-2.697220	-1.658332

Optimized structure of **BTz-TT-FA-3**

Center Number	Atomic Number	Atomic Type	Coordinates (Angstroms) X	Y	Z
1	6	0	-0.671007	-0.415977	-0.005419
2	6	0	0.670842	-0.415951	0.007098
3	6	0	1.426214	0.703343	0.014108
4	6	0	0.679315	1.831172	0.007399
5	6	0	-0.679437	1.831210	-0.006165
6	6	0	-1.426390	0.703434	-0.012800
7	7	0	1.169678	2.999308	0.013025
8	16	0	0.000141	4.230247	0.000412
9	7	0	-1.169617	2.999380	-0.012083
10	6	0	2.785991	0.643307	0.027182
11	6	0	-2.786115	0.643569	-0.026049
12	16	0	3.536916	-0.642614	0.031911
13	6	0	4.900931	-0.132237	0.057152
14	6	0	4.923829	1.190212	0.050903
15	6	0	3.677264	1.661086	0.029890
16	6	0	6.148271	-0.611205	0.096864
17	6	0	7.023532	0.426655	0.173318
18	16	0	6.280513	1.709967	0.040464
19	16	0	-3.537129	-0.642315	-0.030537
20	6	0	-4.901194	-0.131790	-0.056392
21	6	0	-4.923858	1.190667	-0.050500
22	6	0	-3.677273	1.661396	-0.029184
23	6	0	-6.148520	-0.610624	-0.096404
24	6	0	-7.023650	0.427321	-0.173494
25	16	0	-6.280531	1.710615	-0.040680
26	6	0	8.383032	0.394017	0.180387
27	6	0	-8.383215	0.394758	-0.181149
28	6	0	9.120230	-0.635434	0.669908
29	6	0	10.444601	-0.629888	0.468977
30	6	0	11.114734	0.344099	-0.147520
31	6	0	10.427984	1.407871	-0.566704
32	6	0	9.094750	1.410303	-0.380548
33	6	0	8.724259	-1.640705	1.477257
34	6	0	9.558824	-2.610765	1.899961
35	6	0	10.865543	-2.606579	1.585185
36	6	0	11.311145	-1.563483	0.877574
37	6	0	-9.095012	1.411276	0.379159
38	6	0	-10.428338	1.409008	0.564617
39	6	0	-11.115013	0.345375	0.145179
40	6	0	-10.444679	-0.628842	-0.470809
41	6	0	-9.120182	-0.634595	-0.670938
42	6	0	-11.311196	-1.562451	-0.879714
43	6	0	-10.865301	-2.605959	-1.586588
44	6	0	-9.558362	-2.610372	-1.900563
45	6	0	-8.723979	-1.640228	-1.477766
46	6	0	12.410002	-0.001380	-0.169285
47	6	0	12.530771	-1.187100	0.463491
48	6	0	13.719925	-1.789067	0.617311
49	6	0	14.775862	-1.144049	0.099781
50	6	0	14.654318	0.035470	-0.529391
51	6	0	13.468858	0.644046	-0.680624
52	6	0	-14.654908	0.037701	0.524680
53	6	0	-14.776287	-1.142085	-0.104297
54	6	0	-13.720027	-1.788168	-0.619656
55	6	0	-12.531008	-1.185733	-0.466388
56	6	0	-12.410330	0.000052	0.166189
57	6	0	-13.469300	0.645229	0.677957
58	6	0	16.065945	-1.554927	0.134846
59	7	0	16.690274	-0.524349	-0.541020
60	6	0	15.854526	0.493610	-0.958130
61	6	0	-15.854728	0.493665	0.956827
62	7	0	-16.694100	-0.510493	0.514552
63	6	0	-16.065726	-1.555006	-0.135690
64	8	0	16.179989	1.497178	-1.544359
65	8	0	16.595472	-2.532019	0.605503
66	8	0	-16.177431	1.486612	1.562427
67	8	0	-16.592157	-2.543108	-0.586744
68	6	0	18.129744	-0.490163	-0.755515
69	6	0	-18.129294	-0.491464	0.757123
70	6	0	6.405329	-2.071327	-0.196732
71	6	0	-6.405834	-2.070709	0.197407
72	1	0	-1.118100	-1.425021	-0.009514
73	1	0	1.117781	-1.425112	0.011316
74	1	0	3.502144	2.741417	0.015308
75	1	0	-3.502000	2.741670	-0.014858
76	1	0	10.938358	2.244845	-1.069874
77	1	0	8.610027	2.303497	-0.810527
78	1	0	7.696736	-1.672889	1.867369
79	1	0	9.171845	-3.410748	2.554739
80	1	0	11.545944	-3.389674	1.953831
81	1	0	-8.610347	2.304486	0.809263
82	1	0	-10.938835	2.246138	1.067535
83	1	0	-11.545508	-3.388967	-1.955305
84	1	0	-9.171114	-3.410692	-2.554705
85	1	0	-7.696217	-1.672744	-1.867165
86	1	0	13.823480	-2.752045	1.139493
87	1	0	13.370725	1.613838	-1.191024
88	1	0	-13.823120	-2.752393	-1.139435
89	1	0	-13.370839	1.613943	1.190350
90	1	0	18.550391	-1.515600	-0.855812
91	1	0	18.388584	0.052893	-1.691841
92	1	0	18.626350	0.018258	0.101834
93	1	0	-18.523717	0.549112	0.779234
94	1	0	-18.684979	-1.025199	-0.046036
95	1	0	-18.353127	-0.979938	1.732528
96	1	0	5.720146	-2.420034	-1.002627
97	1	0	7.419760	-2.263776	-0.607656
98	1	0	6.200137	-2.721882	0.682349
99	1	0	-5.721182	-2.419205	1.003749
100	1	0	-7.420557	-2.262862	0.607912
101	1	0	-6.200403	-2.721457	-0.681401

Optimized structure of **BTz-TT-FA-4**

Center Number	Atomic Number	Atomic Type	X	Y	Z
1	6	0	0.671394	-0.295226	-0.176031
2	6	0	-0.670004	-0.282246	-0.167230
3	6	0	-1.408477	0.824622	0.045883
4	6	0	-0.654477	1.932845	0.244198
5	6	0	0.704622	1.915626	0.234501
6	6	0	1.438316	0.797841	0.026818
7	7	0	-1.127889	3.090451	0.460773
8	16	0	0.061367	4.283519	0.674736
9	7	0	1.212614	3.056986	0.444036
10	6	0	-2.765806	0.744954	0.047801
11	6	0	2.797366	0.721784	0.016321
12	16	0	-3.694281	1.880699	0.273770
13	6	0	-4.972853	1.186530	0.177466
14	6	0	-4.797278	-0.100547	-0.065326
15	6	0	-3.496581	-0.378017	-0.144220
16	6	0	-6.279260	1.465557	0.252077
17	6	0	-7.007082	0.332741	0.030956
18	16	0	-6.064425	-0.799291	-0.150578
19	16	0	3.529047	-0.553688	-0.218753
20	6	0	4.899864	-0.077490	-0.096050
21	6	0	4.943356	1.224019	0.135905
22	6	0	3.703746	1.709364	0.200298
23	6	0	6.138434	-0.577780	-0.135863
24	6	0	7.029075	0.413585	0.133565
25	16	0	6.308890	1.713674	0.221906
26	6	0	-8.360675	0.154916	0.006033
27	6	0	8.387266	0.352000	0.150702
28	6	0	-9.058877	-1.019854	0.087588
29	6	0	-10.401181	-0.963486	0.009419
30	6	0	-11.121647	0.151030	-0.133593
31	6	0	-10.470418	1.309315	-0.208224
32	6	0	-9.129642	1.264755	-0.139129
33	6	0	-8.642857	-2.292725	0.289349
34	6	0	-9.467096	-3.357612	0.350142
35	6	0	-10.797237	-3.232748	0.232344
36	6	0	-11.259655	-1.991006	0.069178
37	6	0	9.127004	1.435089	-0.213834
38	6	0	10.463087	1.435927	-0.379447
39	6	0	11.122252	0.300654	-0.143109
40	6	0	10.423207	-0.751434	0.284009
41	6	0	9.095759	-0.763161	0.462438
42	6	0	11.264290	-1.760779	0.535465
43	6	0	10.786581	-2.901559	1.043272
44	6	0	9.474666	-2.931982	1.334297
45	6	0	8.666537	-1.884920	1.076154
46	6	0	-12.421249	-0.172879	-0.178252
47	6	0	-12.506816	-1.511350	-0.051288
48	6	0	-13.687554	-2.148669	-0.052014
49	6	0	-14.772810	-1.372819	-0.189130
50	6	0	-14.685939	-0.039268	-0.316320
51	6	0	-13.508001	0.601639	-0.314087
52	6	0	14.661811	-0.013807	-0.520696
53	6	0	14.749541	-1.287567	-0.106134
54	6	0	13.672294	-1.990343	0.273518
55	6	0	12.497998	-1.344491	0.211182
56	6	0	12.410857	-0.064177	-0.206153
57	6	0	13.490912	0.636896	-0.582309
58	6	0	-16.061493	-1.788997	-0.214802
59	7	0	-16.723785	-0.588329	-0.383497
60	6	0	-15.910375	0.527098	-0.435893
61	6	0	15.877538	0.484247	-0.849340
62	7	0	16.689625	-0.599930	-0.578000
63	6	0	16.030190	-1.727885	-0.129265
64	8	0	-16.267426	1.673569	-0.556744
65	8	0	-16.565145	-2.881818	-0.122092
66	8	0	16.229678	1.560555	-1.267052
67	8	0	16.529572	-2.790729	0.149375
68	6	0	-18.175200	-0.500506	-0.452512
69	6	0	18.128986	-0.570559	-0.793787
70	6	0	-6.712547	2.847260	0.687948
71	6	0	6.375511	-1.966086	-0.683973
72	1	0	1.105819	-1.292455	-0.362984
73	1	0	-1.131581	-1.265185	-0.347796
74	1	0	-3.145537	-1.400372	-0.333011
75	1	0	3.543954	2.776440	0.384814
76	1	0	-11.012349	2.257081	-0.357724
77	1	0	-8.693739	2.255272	-0.304486
78	1	0	-7.594903	-2.573331	0.446076
79	1	0	-9.047978	-4.365800	0.517390
80	1	0	-11.465649	-4.105149	0.291842
81	1	0	8.665420	2.399533	-0.486815
82	1	0	10.997829	2.336701	-0.721232
83	1	0	11.445191	-3.751628	1.278807
84	1	0	9.060921	-3.825076	1.833773
85	1	0	7.631805	-1.962333	1.440412
86	1	0	-13.762879	-3.241073	0.054791
87	1	0	-13.437966	1.694962	-0.415126
88	1	0	13.747784	-3.032465	0.618295
89	1	0	13.420035	1.682095	-0.918754
90	1	0	-18.616081	-1.412190	-0.914259
91	1	0	-18.594432	-0.379942	0.572125
92	1	0	-18.501468	0.361635	-1.076160
93	1	0	18.543393	0.449713	-0.632458
94	1	0	18.660251	-1.245376	-0.085902
95	1	0	18.360364	-0.890462	-1.835113
96	1	0	-5.901965	3.379134	1.233845
97	1	0	-6.962003	3.492267	-0.184653
98	1	0	-7.542993	2.814113	1.428132
99	1	0	5.697705	-2.149861	-1.548554
100	1	0	7.393113	-2.101650	-1.109245
101	1	0	6.144750	-2.759985	0.060681

Optimized structure of **BTz-TT-FA-5**

Center Number	Atomic Number	Atomic Type	X	Y	Z
1	6	0	-0.638168	-1.078362	0.177780
2	6	0	0.703190	-1.065968	0.169151
3	6	0	1.442136	0.029500	-0.095544
4	6	0	0.688455	1.123928	-0.360473
5	6	0	-0.670620	1.107461	-0.350587
6	6	0	-1.404381	0.004249	-0.076742
7	7	0	1.161693	2.267943	-0.640513
8	16	0	-0.027434	3.446631	-0.923909
9	7	0	-1.178657	2.235379	-0.623665
10	6	0	2.799645	-0.045829	-0.074019
11	6	0	-2.763358	-0.065717	-0.043635
12	16	0	3.726944	1.082390	-0.341964
13	6	0	5.001359	0.403367	-0.149302
14	6	0	4.832612	-0.875249	0.145530

15	6	0	3.530254	-1.154809	0.189807
16	6	0	6.304157	0.699888	-0.170093
17	6	0	7.018387	-0.404368	0.175264
18	16	0	6.099666	-1.569312	0.301696
19	16	0	-3.497872	-1.322801	0.266235
20	6	0	-4.871581	-0.842805	0.182963
21	6	0	-4.906426	0.442619	-0.120721
22	6	0	-3.668556	0.916419	-0.256821
23	6	0	-6.115377	-1.318596	0.312780
24	6	0	-7.018099	-0.325227	0.067662
25	16	0	-6.269972	0.929836	-0.192079
26	6	0	8.368518	-0.554677	0.231071
27	6	0	-8.382967	-0.360992	0.077694
28	6	0	9.235465	0.451022	0.512912
29	6	0	10.548155	0.225323	0.372718
30	6	0	11.083460	-0.941642	0.012841
31	6	0	10.260290	-1.971647	-0.189609
32	6	0	8.937520	-1.756241	-0.062068
33	6	0	8.972903	1.654233	1.063105
34	6	0	9.928626	2.575171	1.296793
35	6	0	11.225539	2.328169	1.044363
36	6	0	11.530263	1.103997	0.601744
37	6	0	-8.972144	-1.582177	0.002865
38	6	0	-10.290860	-1.836552	-0.025865
39	6	0	-11.112823	-0.790714	0.018188
40	6	0	-10.572160	0.427263	0.093981
41	6	0	-9.253663	0.694067	0.131278
42	6	0	-11.578713	1.311273	0.135170
43	6	0	-11.311981	2.615772	0.233581
44	6	0	-10.015267	2.950044	0.308896
45	6	0	-9.036715	2.023781	0.268772
46	6	0	12.414134	-0.783122	-0.031936
47	6	0	12.690444	0.487159	0.329983
48	6	0	13.949040	0.947069	0.395918
49	6	0	14.911544	0.070480	0.074276
50	6	0	14.635872	-1.192979	-0.285837
51	6	0	13.379957	-1.659225	-0.346957
52	6	0	-14.666192	-1.162557	-0.073924
53	6	0	-14.957409	0.145786	0.000173
54	6	0	-14.003582	1.085676	0.075256
55	6	0	-12.738227	0.639538	0.069700
56	6	0	-12.447733	-0.673936	-0.003726
57	6	0	-13.403051	-1.612692	-0.077153
58	6	0	16.245189	0.306475	0.069313
59	7	0	16.729382	-0.920879	-0.340014
60	6	0	15.766580	-1.887804	-0.556434
61	6	0	-15.789496	-1.916586	-0.136469
62	7	0	-16.765731	-0.939437	-0.108476
63	6	0	-16.295436	0.355640	-0.007638
64	8	0	15.957448	-3.030277	-0.895626
65	8	0	16.898469	1.285802	0.335528
66	8	0	-15.965700	-3.108882	-0.200005
67	8	0	-16.961554	1.360308	0.053336
68	6	0	18.152551	-1.189601	-0.486092
69	6	0	-18.186732	-1.254092	-0.134484
70	6	0	6.772276	2.003845	-0.774164
71	6	0	-6.315441	-2.729035	0.820075
72	1	0	-1.073434	-2.063460	0.419035
73	1	0	1.164386	-2.037566	0.403717
74	1	0	3.177417	-2.168632	0.417475
75	1	0	-3.509763	1.971573	-0.500818
76	1	0	10.655135	-2.959871	-0.475270
77	1	0	8.336315	-2.649313	-0.304299
78	1	0	7.955218	1.908269	1.393410
79	1	0	9.648785	3.544884	1.743330
80	1	0	12.003383	3.076950	1.259292
81	1	0	-8.390790	-2.499784	-0.132785
82	1	0	-10.681539	-2.862571	-0.119485
83	1	0	-12.106625	3.376068	0.275743
84	1	0	-9.754841	4.017337	0.422781
85	1	0	-8.042268	2.469827	0.384821
86	1	0	14.178159	1.979923	0.698115
87	1	0	13.155147	-2.696675	-0.636457
88	1	0	-14.246068	2.156933	0.138283
89	1	0	-13.165559	-2.685471	-0.134732
90	1	0	18.709927	-0.275283	-0.789561
91	1	0	18.567438	-1.557694	0.479628
92	1	0	18.343352	-1.954117	-1.272156
93	1	0	-18.387126	-2.184121	-0.711905
94	1	0	-18.561262	-1.390985	0.905249
95	1	0	-18.773426	-0.443958	-0.622482
96	1	0	6.153370	2.248756	-1.667292
97	1	0	6.653412	2.860990	-0.074686
98	1	0	7.808487	1.955702	-1.172930
99	1	0	-5.418009	-3.102546	1.361351
100	1	0	-7.122189	-2.791146	1.584248
101	1	0	-6.482633	-3.444676	-0.016350

Optimized structure of **BTz-TT-FA-6**

Center Number	Atomic Number	Atomic Type	Coordinates (Angstroms) X	Y	Z
1	6	0	0.670719	-0.297310	-0.217898
2	6	0	-0.670779	-0.284000	-0.209151
3	6	0	-1.408790	0.821241	0.014001
4	6	0	-0.654429	1.927637	0.220831
5	6	0	0.704672	1.909895	0.211292
6	6	0	1.437906	0.793510	-0.004934
7	7	0	-1.127381	3.083683	0.446833
8	16	0	0.062133	4.274670	0.670479
9	7	0	1.212924	3.049543	0.430179
10	6	0	-2.766114	0.741985	0.017013
11	6	0	2.797064	0.717114	-0.013875
12	16	0	-3.693626	1.876425	0.253942
13	6	0	-4.972666	1.183290	0.157016
14	6	0	-4.798247	-0.101685	-0.097054
15	6	0	-3.497847	-0.379007	-0.182452
16	6	0	-6.278573	1.462042	0.239341
17	6	0	-7.007649	0.331233	0.012266
18	16	0	-6.065963	-0.799340	-0.183439
19	16	0	3.529489	-0.556002	-0.259402
20	6	0	4.899900	-0.081331	-0.126384
21	6	0	4.942650	1.217862	0.117999
22	6	0	3.702817	1.702802	0.182239
23	6	0	6.138995	-0.580913	-0.165287
24	6	0	7.028310	0.407715	0.118027
25	16	0	6.307580	1.706958	0.214486
26	6	0	-8.361421	0.153974	-0.006020
27	6	0	8.386418	0.346821	0.143594
28	6	0	-9.059483	-1.021202	0.070918
29	6	0	-10.402366	-0.963371	0.003628
30	6	0	-11.123050	0.152647	-0.124762
31	6	0	-10.471864	1.311038	-0.196392
32	6	0	-9.130707	1.265151	-0.137762
33	6	0	-8.642739	-2.295922	0.258902
34	6	0	-9.467238	-3.360767	0.317432

35	6	0	-10.798259	-3.234021	0.211607
36	6	0	-11.261200	-1.990689	0.062275
37	6	0	9.127850	1.434348	-0.204250
38	6	0	10.465156	1.438472	-0.359557
39	6	0	11.123850	0.301680	-0.129871
40	6	0	10.422809	-0.755639	0.280746
41	6	0	9.093937	-0.770779	0.448757
42	6	0	11.263220	-1.766480	0.528362
43	6	0	10.783316	-2.913068	1.020360
44	6	0	9.469275	-2.948296	1.300805
45	6	0	8.661718	-1.899572	1.047556
46	6	0	-12.423320	-0.169938	-0.160752
47	6	0	-12.509051	-1.509324	-0.043428
48	6	0	-13.690552	-2.145221	-0.038500
49	6	0	-14.776208	-1.367285	-0.159586
50	6	0	-14.688873	-0.032918	-0.277140
51	6	0	-13.510273	0.606759	-0.280666
52	6	0	14.667081	-0.003822	-0.480538
53	6	0	14.753132	-1.281600	-0.078062
54	6	0	13.673574	-1.989896	0.284757
55	6	0	12.498946	-1.344953	0.218664
56	6	0	12.413585	-0.060594	-0.186176
57	6	0	13.495650	0.645871	-0.545895
58	6	0	-16.065556	-1.781559	-0.176571
59	7	0	-16.727868	-0.578813	-0.329931
60	6	0	-15.913601	0.535921	-0.380649
61	6	0	15.885026	0.499359	-0.792957
62	7	0	16.696345	-0.586208	-0.524885
63	6	0	16.034513	-1.719688	-0.093860
64	8	0	-16.270276	1.683859	-0.488916
65	8	0	-16.569971	-2.874548	-0.087449
66	8	0	16.239258	1.580340	-1.196518
67	8	0	16.533021	-2.784599	0.178767
68	6	0	-18.179760	-0.488495	-0.385274
69	6	0	18.137536	-0.552348	-0.726372
70	6	0	-6.709709	2.839971	0.689408
71	6	0	6.378829	-1.964128	-0.725078
72	1	0	1.104880	-1.292980	-0.413123
73	1	0	-1.133022	-1.265023	-0.397921
74	1	0	-3.147667	-1.400031	-0.380044
75	1	0	3.542991	2.768033	0.376970
76	1	0	-11.014210	2.260255	-0.334633
77	1	0	-8.695335	2.256581	-0.299130
78	1	0	-7.593798	-2.578613	0.404702
79	1	0	-9.047703	-4.370566	0.472825
80	1	0	-11.466838	-4.106451	0.269564
81	1	0	8.667546	2.401311	-0.470299
82	1	0	11.001495	2.343509	-0.687350
83	1	0	11.441349	-3.764609	1.252405
84	1	0	9.053030	-3.847124	1.787776
85	1	0	7.624664	-1.982176	1.403388
86	1	0	-13.766146	-3.238332	0.060457
87	1	0	-13.439968	1.700790	-0.373655
88	1	0	13.747780	-3.035216	0.619786
89	1	0	13.426257	1.694382	-0.872094
90	1	0	-18.626190	-1.396192	-0.849279
91	1	0	-18.589526	-0.374065	0.643872
92	1	0	-18.510028	0.378451	-1.000030
93	1	0	18.548822	0.466542	-0.549289
94	1	0	18.663104	-1.234497	-0.021311
95	1	0	18.379457	-0.859874	-1.769007
96	1	0	-5.896469	3.367543	1.235278
97	1	0	-6.963851	3.492297	-0.176365
98	1	0	-7.536170	2.800418	1.433685
99	1	0	5.706448	-2.139128	-1.595704
100	1	0	7.399161	-2.096468	-1.145262
101	1	0	6.142962	-2.765082	0.010398

Optimized structure of **BTz-TT-FA-7**

Center Number	Atomic Number	Atomic Type	Coordinates (Angstroms) X	Y	Z
1	6	0	-0.714205	-0.645976	0.252761
2	6	0	0.626512	-0.683849	0.280750
3	6	0	1.413851	0.362310	-0.038175
4	6	0	0.709787	1.465066	-0.390717
5	6	0	-0.648724	1.499416	-0.417978
6	6	0	-1.431530	0.443442	-0.097269
7	7	0	1.234000	2.570133	-0.730355
8	16	0	0.099267	3.771538	-1.120695
9	7	0	-1.105152	2.625289	-0.777054
10	6	0	2.766687	0.235482	0.015577
11	6	0	-2.792613	0.422271	-0.109244
12	16	0	3.742908	1.305787	-0.309354
13	6	0	4.986096	0.586244	-0.066760
14	6	0	4.761101	-0.661651	0.310488
15	6	0	3.447666	-0.882552	0.361310
16	6	0	6.300805	0.823535	-0.103381
17	6	0	6.966100	-0.286801	0.313251
18	16	0	5.996287	-1.397557	0.520465
19	16	0	-3.581975	-0.785769	0.258658
20	6	0	-4.929582	-0.269458	0.061923
21	6	0	-4.913006	0.995523	-0.323929
22	6	0	-3.652892	1.417430	-0.425400
23	6	0	-6.189986	-0.706496	0.145771
24	6	0	-7.033430	0.284496	-0.248556
25	16	0	-6.254117	1.530326	-0.487687
26	6	0	8.308671	-0.494331	0.369893
27	6	0	-8.393117	0.284664	-0.267932
28	6	0	9.223039	0.487344	0.576462
29	6	0	10.522282	0.194395	0.435888
30	6	0	11.000193	-1.016336	0.146753
31	6	0	10.129736	-2.019518	0.023372
32	6	0	8.819543	-1.737044	0.151514
33	6	0	9.021174	1.734307	1.049164
34	6	0	10.019878	2.624619	1.210532
35	6	0	11.300796	2.304235	0.958894
36	6	0	11.545372	1.041594	0.593764
37	6	0	-9.080733	1.437981	-0.042455
38	6	0	-10.415122	1.522182	0.114911
39	6	0	-11.127511	0.398965	0.016042
40	6	0	-10.479563	-0.729052	-0.276530
41	6	0	-9.154241	-0.825169	-0.445538
42	6	0	-11.367898	-1.720605	-0.406822
43	6	0	-10.945356	-2.935896	-0.769933
44	6	0	-9.636614	-3.063537	-1.048601
45	6	0	-8.779131	-2.032436	-0.915646
46	6	0	12.335543	-0.920316	0.073163
47	6	0	12.672876	0.357333	0.346922
48	6	0	13.951258	0.764097	0.363493
49	6	0	14.869044	-0.173184	0.085111
50	6	0	14.532465	-1.443969	-0.187057
51	6	0	13.256675	-1.857008	-0.198073
52	6	0	-14.677252	0.300145	0.416013
53	6	0	-14.825797	-1.009035	0.158932
54	6	0	-13.783604	-1.802653	-0.128453

55	6	0	-12.580007	-1.210131	-0.140773
56	6	0	-12.431912	0.105924	0.117905
57	6	0	-13.476721	0.897694	0.402162
58	6	0	16.211336	0.002579	0.043466
59	7	0	16.634283	-1.268952	-0.292493
60	6	0	15.626750	-2.204269	-0.429573
61	6	0	-15.867478	0.891159	0.677506
62	7	0	-16.730335	-0.178552	0.536698
63	6	0	-16.125626	-1.382419	0.230663
64	8	0	15.761747	-3.373547	-0.696313
65	8	0	16.911316	0.967064	0.234292
66	8	0	-16.167651	2.025412	0.960199
67	8	0	-16.675208	-2.446585	0.081278
68	6	0	18.041507	-1.609166	-0.444235
69	6	0	-18.166158	-0.055552	0.741994
70	6	0	6.824322	2.062158	-0.793580
71	6	0	-6.492308	-2.007417	0.853313
72	1	0	-1.192882	-1.596647	0.544704
73	1	0	1.043780	-1.654326	0.590361
74	1	0	3.050203	-1.864412	0.648062
75	1	0	-3.445337	2.448111	-0.729794
76	1	0	10.476554	-3.041019	-0.201524
77	1	0	8.176341	-2.616222	-0.025131
78	1	0	8.020119	2.054286	1.373052
79	1	0	9.789409	3.633116	1.595408
80	1	0	12.113743	3.030113	1.114108
81	1	0	-8.574441	2.406042	0.113572
82	1	0	-10.906220	2.482242	0.341547
83	1	0	-11.643839	-3.776221	-0.903455
84	1	0	-9.266414	-4.029518	-1.433544
85	1	0	-7.749551	-2.202430	-1.262308
86	1	0	14.230244	1.803345	0.593271
87	1	0	12.981899	-2.899910	-0.416000
88	1	0	-13.908862	-2.874296	-0.344170
89	1	0	-13.356078	1.971477	0.609430
90	1	0	18.631555	-0.742197	-0.817040
91	1	0	18.459182	-1.931191	0.536586
92	1	0	18.183880	-2.431138	-1.180917
93	1	0	-18.532613	0.955475	0.455116
94	1	0	-18.730176	-0.786426	0.120456
95	1	0	-18.410185	-0.233867	1.813861
96	1	0	6.210590	2.278630	-1.697394
97	1	0	6.751787	2.967007	-0.150222
98	1	0	7.853558	1.939226	-1.194118
99	1	0	-5.824337	-2.119727	1.737480
100	1	0	-7.515363	-2.043609	1.285847
101	1	0	-6.299361	-2.893860	0.209048

Optimized structure of **BTz-TT-FA-8**

Center Number	Atomic Number	Atomic Type	Coordinates (Angstroms) X	Y	Z
1	6	0	0.670724	-0.223239	-0.146977
2	6	0	-0.670479	-0.223223	-0.147526
3	6	0	-1.420652	0.886849	-0.001449
4	6	0	-0.679794	2.013394	0.136585
5	6	0	0.679809	2.013346	0.137331
6	6	0	1.420753	0.886719	-0.000190
7	7	0	-1.170770	3.174677	0.282213
8	16	0	-0.000131	4.395135	0.434104
9	7	0	1.170634	3.174687	0.283401
10	6	0	-2.777234	0.791896	-0.001146
11	6	0	2.777277	0.791876	0.001202
12	16	0	-3.721359	1.926515	0.156609
13	6	0	-4.991122	1.212142	0.099033
14	6	0	-4.798206	-0.084068	-0.070961
15	6	0	-3.493817	-0.348883	-0.131769
16	6	0	-6.301111	1.477141	0.158139
17	6	0	-7.013624	0.323856	0.001503
18	16	0	-6.055576	-0.803412	-0.118563
19	16	0	3.721456	1.926544	0.159262
20	6	0	4.991097	1.212095	0.101923
21	6	0	4.798312	-0.084085	-0.067770
22	6	0	3.493996	-0.348993	-0.128895
23	6	0	6.301213	1.477259	0.160842
24	6	0	7.013703	0.323999	0.004733
25	16	0	6.055746	-0.803455	-0.114829
26	6	0	-8.364603	0.125391	-0.010617
27	6	0	8.364628	0.125751	-0.007197
28	6	0	-9.045493	-1.053227	0.136160
29	6	0	-10.388751	-1.020252	0.060299
30	6	0	-11.125434	0.074722	-0.139137
31	6	0	-10.491374	1.236328	-0.279241
32	6	0	-9.149897	1.214710	-0.213055
33	6	0	-8.610897	-2.307711	0.403717
34	6	0	-9.419691	-3.379371	0.524593
35	6	0	-10.751833	-3.279662	0.405906
36	6	0	-11.232377	-2.054979	0.178721
37	6	0	9.149837	1.214894	-0.210843
38	6	0	10.491265	1.236556	-0.277432
39	6	0	11.125508	0.075080	-0.136161
40	6	0	10.388888	-1.019596	0.064856
41	6	0	9.045641	-1.052711	0.140921
42	6	0	11.232666	-2.054187	0.184134
43	6	0	10.752209	-3.278637	0.412867
44	6	0	9.420046	-3.378326	0.531851
45	6	0	8.611238	-2.306836	0.410000
46	6	0	-12.420490	-0.269303	-0.160252
47	6	0	-12.486754	-1.600102	0.038592
48	6	0	-13.658378	-2.252675	0.077860
49	6	0	-14.755101	-1.500361	-0.095152
50	6	0	-14.687346	-0.174564	-0.293920
51	6	0	-13.518515	0.481831	-0.332045
52	6	0	14.687308	-0.174023	-0.291410
53	6	0	14.755254	-1.499654	-0.091280
54	6	0	13.658573	-2.252144	0.081408
55	6	0	12.486894	-1.599345	0.043150
56	6	0	12.420557	-0.268820	-0.157256
57	6	0	13.518434	0.481944	-0.331417
58	6	0	-16.037922	-1.934847	-0.091887
59	7	0	-16.717650	-0.754128	-0.321250
60	6	0	-15.920059	0.367836	-0.437090
61	6	0	15.919697	0.367544	-0.440613
62	7	0	16.718825	-0.749979	-0.295555
63	6	0	16.037836	-1.934748	-0.093077
64	8	0	-16.293563	1.501328	-0.617006
65	8	0	-16.526149	-3.027912	0.061835
66	8	0	16.291982	1.497367	-0.644471
67	8	0	16.524999	-3.031180	0.038939
68	6	0	-18.170347	-0.689897	-0.387558
69	6	0	18.169816	-0.690629	-0.395552
70	6	0	-6.752891	2.875315	0.515868
71	6	0	6.753135	2.875526	0.518232
72	1	0	1.120615	-1.219741	-0.273906
73	1	0	-1.120289	-1.219835	-0.274719
74	1	0	-3.130384	-1.376164	-0.261306
75	1	0	3.130627	-1.376307	-0.258441

76	1	0	-11.047366	2.166862	-0.477316
77	1	0	-8.728787	2.200977	-0.433038
78	1	0	-7.558742	-2.565576	0.570596
79	1	0	-8.985811	-4.371266	0.743438
80	1	0	-11.407564	-4.156972	0.514675
81	1	0	8.728644	2.200836	-0.431601
82	1	0	11.047174	2.166880	-0.476595
83	1	0	11.408081	-4.155701	0.522590
84	1	0	8.986326	-4.369967	0.752019
85	1	0	7.559089	-2.564429	0.577525
86	1	0	-13.718188	-3.338764	0.243465
87	1	0	-13.464301	1.569022	-0.491665
88	1	0	13.718163	-3.338383	0.245384
89	1	0	13.463946	1.568581	-0.494901
90	1	0	-18.600217	-1.630285	-0.799546
91	1	0	-18.586794	-0.522342	0.631714
92	1	0	-18.511447	0.134289	-1.053020
93	1	0	18.562445	0.287601	-0.038137
94	1	0	18.652783	-1.474593	0.229556
95	1	0	18.480796	-0.835597	-1.455045
96	1	0	-5.949087	3.449286	1.028160
97	1	0	-7.012889	3.465627	-0.391676
98	1	0	-7.581423	2.873211	1.258903
99	1	0	5.949036	3.450004	1.029237
100	1	0	7.580905	2.873018	1.262141
101	1	0	7.014337	3.465460	-0.389142

Optimized structure of **BTz-TT-FA-9**

Center Number	Atomic Number	Atomic Type	Coordinates (Angstroms) X	Y	Z
1	6	0	0.624594	-0.633587	0.234405
2	6	0	-0.715409	-0.580564	0.204480
3	6	0	-1.415822	0.539399	-0.062816
4	6	0	-0.626307	1.616387	-0.294620
5	6	0	0.731994	1.562522	-0.264571
6	6	0	1.422754	0.426845	0.000212
7	7	0	-1.065619	2.776403	-0.563397
8	16	0	0.157076	3.928901	-0.807750
9	7	0	1.272873	2.683819	-0.511630
10	6	0	-2.775094	0.500652	-0.078992
11	6	0	2.774201	0.280745	0.045365
12	16	0	-3.666833	1.653407	-0.360339
13	6	0	-4.967039	1.003634	-0.248418
14	6	0	-4.833295	-0.277622	0.045982
15	6	0	-3.542353	-0.591027	0.148664
16	6	0	-6.263322	1.319783	-0.344972
17	6	0	-7.027529	0.219713	-0.085336
18	16	0	-6.122174	-0.933571	0.146319
19	16	0	3.764963	1.359961	-0.195816
20	6	0	4.999110	0.608170	-0.011839
21	6	0	4.757501	-0.661621	0.269670
22	6	0	3.441402	-0.868763	0.304337
23	6	0	6.316766	0.829638	-0.033922
24	6	0	6.967479	-0.318002	0.295322
25	16	0	5.982733	-1.427894	0.421356
26	6	0	-8.386204	0.086354	-0.063902
27	6	0	-9.121476	-1.067649	-0.106857
28	6	0	-10.461790	-0.964844	-0.042194
29	6	0	-11.146253	0.177098	0.052957
30	6	0	-10.458367	1.315764	0.090697
31	6	0	-9.119419	1.225212	0.034238
32	6	0	-8.745662	-2.359981	-0.256721
33	6	0	-9.604000	-3.399045	-0.284841
34	6	0	-10.930243	-3.226624	-0.183669
35	6	0	-11.353130	-1.965348	-0.071083
36	6	0	18.021143	-1.743504	-0.588691
37	8	0	16.930344	0.792236	0.279701
38	8	0	15.714868	-3.450749	-0.964672
39	6	0	16.215771	-0.145217	0.020366
40	7	0	16.619267	-1.394720	-0.409261
41	6	0	15.597752	-2.302396	-0.612247
42	6	0	-12.456116	-0.102470	0.098195
43	6	0	-12.584824	-1.441180	0.020169
44	6	0	-13.786068	-2.038804	0.033890
45	6	0	-14.846072	-1.223143	0.132226
46	6	0	-14.715989	0.110701	0.210753
47	6	0	-13.517434	0.712049	0.195386
48	6	0	13.233406	-1.938091	-0.349425
49	6	0	12.326921	-1.010450	-0.007219
50	6	0	12.683624	0.238353	0.358993
51	6	0	13.967950	0.623982	0.401868
52	6	0	14.871169	-0.303784	0.052558
53	6	0	14.515276	-1.545909	-0.311584
54	6	0	9.054357	1.613027	1.172750
55	6	0	10.066513	2.474189	1.396396
56	6	0	11.342001	2.154600	1.117552
57	6	0	-16.147938	-1.595233	0.161596
58	7	0	-16.771321	-0.367949	0.279672
59	6	0	-15.921712	0.721228	0.297928
60	6	0	11.566850	0.918952	0.659269
61	8	0	-16.241405	1.882630	0.372716
62	8	0	-16.687074	-2.673618	0.104736
63	6	0	9.236374	0.401671	0.608159
64	6	0	10.530991	0.101048	0.442230
65	6	0	10.990344	-1.091876	0.062801
66	6	0	10.104831	-2.070145	-0.132239
67	6	0	-18.219468	-0.229559	0.332009
68	6	0	8.799378	-1.778660	0.020235
69	6	0	8.307129	-0.548317	0.332094
70	6	0	-6.650094	2.695946	-0.838625
71	6	0	6.855829	2.109738	-0.630351
72	1	0	1.030263	-1.629710	0.468048
73	1	0	-1.208976	-1.541054	0.417597
74	1	0	-3.225054	-1.615373	0.381668
75	1	0	3.032069	-1.865039	0.514915
76	1	0	-10.969994	2.285464	0.200710
77	1	0	-8.652380	2.206428	0.167001
78	1	0	-7.706209	-2.679905	-0.393223
79	1	0	-9.217050	-4.425880	-0.410190
80	1	0	-11.626305	-4.078682	-0.216513
81	1	0	18.622825	-0.860863	-0.901079
82	1	0	18.435917	-2.139714	0.365945
83	1	0	18.150410	-2.513593	-1.381830
84	1	0	-13.896817	-3.131385	-0.032929
85	1	0	-13.411960	1.805535	0.256450
86	1	0	12.942681	-2.957999	-0.642785
87	1	0	14.262935	1.639122	0.706835
88	1	0	8.058833	1.922837	1.522819
89	1	0	9.851783	3.454676	1.855553
90	1	0	12.165982	2.854990	1.323521
91	1	0	10.436000	-3.077075	-0.433300
92	1	0	-18.694827	-1.110062	0.819338
93	1	0	-18.521365	0.663382	0.923738
94	1	0	-18.625898	-0.129017	-0.699948
95	1	0	8.143065	-2.632899	-0.219457
96	1	0	-5.820422	3.179415	-1.400556

97	1	0	-7.477804	2.659560	-1.581613
98	1	0	-6.882740	3.383304	0.005952
99	1	0	6.243050	2.402245	-1.513163
100	1	0	6.797285	2.964347	0.079672
101	1	0	7.882327	2.003333	-1.042727

Optimized structure of **BTz-TT-FA-10**

Center	Atomic	Atomic	Coordinates (Angstroms)		
Number	Number	Type	X	Y	Z
1	6	0	-0.670602	-0.998677	-0.003891
2	6	0	0.670546	-0.998551	0.004646
3	6	0	1.420869	0.121043	0.008196
4	6	0	0.679777	1.255834	0.003909
5	6	0	-0.679966	1.255749	-0.003153
6	6	0	-1.420924	0.120834	-0.007540
7	7	0	1.170403	2.426385	0.006896
8	16	0	-0.000198	3.656197	0.000523
9	7	0	-1.170773	2.426203	-0.005942
10	6	0	2.777617	0.027035	0.017074
11	6	0	-2.777676	0.026742	-0.016510
12	16	0	3.720784	1.173916	0.016752
13	6	0	4.986453	0.452802	0.042322
14	6	0	4.799832	-0.856801	0.039426
15	6	0	3.493877	-1.122003	0.021161
16	6	0	6.293608	0.728203	0.080183
17	6	0	6.992742	-0.435294	0.160439
18	16	0	6.056543	-1.585797	0.029633
19	16	0	-3.720926	1.173567	-0.016288
20	6	0	-4.986589	0.452277	-0.042195
21	6	0	-4.799783	-0.857326	-0.039284
22	6	0	-3.493863	-1.122325	-0.020795
23	6	0	-6.293765	0.727578	-0.080380
24	6	0	-6.992788	-0.435931	-0.160833
25	16	0	-6.056521	-1.586433	-0.029726
26	6	0	8.340603	-0.615084	0.168557
27	6	0	-8.340622	-0.615842	-0.169435
28	6	0	9.227507	0.288343	0.658274
29	6	0	10.535070	0.078429	0.457952
30	6	0	11.047028	-0.987804	-0.157553
31	6	0	10.204320	-1.933321	-0.575744
32	6	0	8.886483	-1.729986	-0.390482
33	6	0	8.990692	1.342995	1.465081
34	6	0	9.964472	2.173432	1.887453
35	6	0	11.254987	1.967949	1.572899
36	6	0	11.534705	0.868009	0.866223
37	6	0	-8.886483	-1.730745	0.389401
38	6	0	-10.204394	-1.934411	0.574182
39	6	0	-11.047151	-0.989124	0.155508
40	6	0	-10.535120	0.077248	-0.459792
41	6	0	-9.227454	0.287535	-0.659533
42	6	0	-11.534672	0.866771	-0.868433
43	6	0	-11.254835	1.966837	-1.574806
44	6	0	-9.964236	2.172554	-1.888779
45	6	0	-8.990516	1.342287	-1.466133
46	6	0	12.380060	-0.845562	-0.179190
47	6	0	12.681763	0.308127	0.452557
48	6	0	13.949074	0.720819	0.605972
49	6	0	14.893302	-0.078934	0.089127
50	6	0	14.592327	-1.226624	-0.538824
51	6	0	13.327611	-1.646222	-0.689749
52	6	0	-14.592483	-1.228604	0.534899
53	6	0	-14.893538	-0.080989	-0.093252
54	6	0	-13.949071	0.719661	-0.608269
55	6	0	-12.681832	0.306551	-0.455317
56	6	0	-12.380176	-0.847065	0.176532
57	6	0	-13.327657	-1.647309	0.687761
58	6	0	16.231102	0.129501	0.123868
59	7	0	16.690118	-0.985221	-0.550924
60	6	0	15.708262	-1.863641	-0.966862
61	6	0	-15.708229	-1.863391	0.966593
62	7	0	-16.691652	-0.999304	0.525184
63	6	0	-16.231024	0.129577	-0.124213
64	8	0	15.875790	-2.905933	-1.551790
65	8	0	16.904184	1.014385	0.593541
66	8	0	-15.875023	-2.894504	1.571310
67	8	0	-16.902510	1.025794	-0.574471
68	6	0	18.107332	-1.239071	-0.765182
69	6	0	-18.106908	-1.238158	0.768056
70	6	0	6.778421	2.128351	-0.218420
71	6	0	-6.778674	2.127736	0.218200
72	1	0	-1.120074	-2.003415	-0.007249
73	1	0	1.120110	-2.003317	0.007992
74	1	0	3.128713	-2.156723	0.010767
75	1	0	-3.128536	-2.157010	-0.010410
76	1	0	10.579472	-2.839654	-1.077909
77	1	0	8.268729	-2.537386	-0.819679
78	1	0	7.980148	1.533383	1.854617
79	1	0	9.705108	3.023825	2.541458
80	1	0	12.047702	2.637270	1.940902
81	1	0	-8.268890	-2.538148	0.818871
82	1	0	-10.579633	-2.840682	1.076227
83	1	0	-12.047581	2.636118	-1.943069
84	1	0	-9.704825	3.023165	-2.542559
85	1	0	-7.979753	1.532900	-1.855187
86	1	0	14.199168	1.657013	1.127235
87	1	0	13.081747	-2.589924	-1.199428
88	1	0	-14.198840	1.657071	-1.127416
89	1	0	-13.081828	-2.589711	1.199624
90	1	0	18.678035	-0.289798	-0.874607
91	1	0	18.522462	-1.809017	0.096890
92	1	0	18.280157	-1.823392	-1.696455
93	1	0	-18.337770	-2.326765	0.786528
94	1	0	-18.401848	-0.792973	1.745324
95	1	0	-18.738462	-0.792546	-0.032844
96	1	0	6.157645	2.577540	-1.026764
97	1	0	6.677579	2.806715	0.657860
98	1	0	7.810853	2.156816	-0.628454
99	1	0	-6.158171	2.576850	1.026705
100	1	0	-6.677722	2.806075	-0.657941
101	1	0	-7.811285	2.156072	0.628032
